# Strengthening Executive Function and Self-Regulation Through Teacher-Student Interaction in Preschool and Primary School Children: A Systematic Review

**DOI:** 10.3389/fpsyg.2021.718262

**Published:** 2021-08-19

**Authors:** Simona Sankalaite, Mariëtte Huizinga, Jolien Dewandeleer, Canmei Xu, Nicky de Vries, Emma Hens, Dieter Baeyens

**Affiliations:** ^1^Parenting and Special Education Research Unit, Faculty of Psychology and Educational Sciences, Katholieke Universiteit Leuven, Leuven, Belgium; ^2^Department of Educational and Family Studies, Faculty of Behavioural and Movement Sciences, Vrije Universiteit Amsterdam, Amsterdam, Netherlands

**Keywords:** executive function, self-regulation, teacher-student interaction, preschool, primary school, intervention

## Abstract

Executive functions (EF) and self-regulation (SR) are fundamental for children's learning, school functioning and academic achievement. EF/SR fail to develop to its full potential if contextual stimulation is not adequately presented. This is evident in the training programmes directly and exclusively targeting EF/SR stimulation, which lack durable and transferable effects. Therefore, recent research has shifted the attention towards malleable environmental factors; more specifically, to the role of school and classroom environment as an important developmental context for promoting children's EF/SR skills and, in turn, their cognition and behaviour. Numerous observational studies have shown a correlation between the quality of teacher-student relationship (TSR) at the dyadic level or teacher-student interaction (TSI) at the classroom level and children's EF/SR skills. To explore the direction of this association, the objective of this systematic literature review was to examine the causal effect of experiments and interventions that aim to improve children's EF/SR by manipulating the TSI. Overall, the results from 18 included studies indicated that children in treatment groups show higher gains, albeit small-sized, in EF/SR performance compared to controls. Furthermore, TSI manipulation seemed to affect children's SR skills more strongly than children's EF skills. More importantly, the findings revealed the largest effects of these manipulations in children considered vulnerable or disadvantaged, suggesting that the cognitive deficits can be minimised if these children are supported appropriately. Given high study heterogeneity, this review highlights the need for more research (and interventions) explicitly investigating TSI and TSR and their potential impact on EF and SR in children. This study aims to provide information as to which specific aspects need to be examined more closely, instructing further development and implementation of efficient and effective interventions in education.

## Introduction

There has been a great interest in supporting and improving children's goal-directed behaviour, guided and controlled by multiple cognitive processes. There are numerous conceptualisations of the cognitive processes involved (e.g., effortful control, cognitive control, self-control), somewhat depending on a particular branch in the field of psychology, with executive functions (EF) and self-regulation (SR) being the most dominant terms used in the literature. EF is an umbrella term describing various cognitive processes that are required to carry out conscious goal-directed behaviours and are especially important in novel and demanding situations, which require a rapid and flexible adjustment of behaviour to the changing demands of the environment (Huizinga et al., 2006; Diamond, 2013). EF is viewed as a construct with partially dissociable components including, but not limited to, three core functions: working memory, inhibition, and cognitive flexibility (e.g., Carlson et al., 2013; Diamond, 2013; Meuwissen and Zelazo, 2014; for a review see Karr et al., 2018). SR is a broad term describing a set of distinct self-initiated behaviours that aim to control and regulate thoughts, feelings and actions (McClelland and Cameron, 2011; e.g., Blair and Raver, 2012), and is usually separated into attentional, emotional, and behavioural regulation, respectively. These, otherwise interchangeable and highly overlapping terms (for a contrasting opinion, see Nigg, 2016), are used depending on the scientific domain, with EF more commonly referred to in cognitive and developmental psychology and SR more prominent in social and personality psychology literature (Hofmann et al., 2012). The use of different definitions to refer to these overlapping cognitive processes leads to poor communication between the educators and the researchers working from different perspectives, lack of reliable, and valid measures being developed and used, scarcity in exchange of the insights, and partial conclusions, which, in turn, slows down efficient and effective intervention development (Bell and Meza, 2020).

It is well documented that EF and SR are fundamental for children's learning, school functioning and academic achievement (Huizinga et al., 2018; for reviews, see McClelland and Cameron, 2011 and Cortés Pascual et al., 2019). EF/SR skills are associated with better school adjustment, as seen in measures of academic ability (Cantin et al., 2016; Mägi et al., 2016) and teachers' perceptions of readiness for learning (Pagani and Fitzpatrick, 2013; Raver et al., 2013; Shaul and Schwartz, 2013; Blair and Raver, 2015), while deficits in EF/SR skills have shown to adversely affect school success, negatively impact further cognitive and social development, and strongly predict behavioural problems both within the classroom and home setting (Jacobson et al., 2011; Lonigan et al., 2017; Morgan et al., 2018). Furthermore, atypical EF/SR development is implicated in a wide range of clinical conditions that affect school functioning, including, but not limited to, attention-deficit/hyperactivity disorder (ADHD) (e.g., Petrovic and Castellanos, 2016), autism spectrum disorder (ASD) (e.g., Zimmerman et al., 2016) and learning disabilities (e.g., Toll et al., 2010).

Numerous studies consistently show that core EF and SR skills rapidly develop in preschool (Garon et al., 2008; Best et al., 2009; Best and Miller, 2010; Montroy et al., 2016) and continue to develop throughout childhood (Davidson et al., 2006; Huizinga et al., 2006; Cameron Ponitz et al., 2008; Macdonald et al., 2013; Chang et al., 2014), with some domains continuing to mature throughout adolescence, and even early adulthood (Luciana et al., 2005; Gross, 2015; Friedman et al., 2016). Neuroimaging research and studies on patients with brain damage (e.g., Aron et al., 2003; Lie et al., 2006) suggest that such EF/SR development mirrors structural and functional changes in brain structures, most commonly linked to the maturation of the prefrontal cortex (Zelazo and Carlson, 2012; Shanmugan and Satterthwaite, 2016; McKenna et al., 2017). However, in general, EF/SR fail to develop to their full potential if the contextual stimulation is not adequately presented (e.g., Fay-Stammbach et al., 2014; Moriguchi, 2014; van Lier and Deater-Deckard, 2015; Helm et al., 2019). Taken into account results from both behavioural and brain imaging studies, early and middle childhood appears to be a period of high plasticity sensitive to developmental and environmental influences (McEwen and Morrison, 2013; Buttelmann and Karbach, 2017; Zelazo and Carlson, 2020).

Given the importance of EF/SR to children's academic outcomes, social and emotional development, and its predictive value of adjustment later in life, there have been numerous attempts to improve EF/SR functioning in preschool and primary school children (e.g., Melby-Lervåg and Hulme, 2013). Research suggests a number of diverse practises and programmes for effectively increasing children's EF/SR (Diamond and Lee, 2011). These effective approaches include direct and indirect approaches to improving EF/SR, as well as a number of educational practises designed to promote EF/SR (Zelazo et al., 2016). Direct approaches can be defined as training involving repeated practise on the increasingly challenging version of a specific type of EF/SR task with the expectation that performance will improve on that task and similar tasks, and transfer to real-world behaviours. Indirect approaches include various activities designed to improve EF/SR indirectly, including exercise and sports, mindfulness practises, and computer games. Most studies aim to improve EF/SR directly; however, although less extensively studied, the literature suggests that indirect approaches may also be highly effective, especially when complementing direct training. Finally, educational practises include classroom curricula (e.g., Tools of the Mind programme; Bodrova and Leong, 2007) and add-ons to classroom curriculum (e.g., Promoting Alternative Thinking Skills (PATHS; Greenberg et al., 1995) and the Chicago School Readiness Project (CSRP; Raver et al., 2009). Such educational practises report promising, however mixed, results regarding EF/SR performance and subsequent academic outcomes.

Despite methodological differences (in duration, setting, activities implemented, and materials/measures used), various interventions have shown promising results for enhancing EF/SR development throughout preschool and primary school (see a review by Diamond and Ling, 2016). Generally, findings indicate that children with initially poorest EF/SR skills show the most improvement from any programme. Early EF/SR interventions focusing on preschoolers can act as prevention focusing on children with a potential delay or impairment in developing such abilities, including children from low socio-economic backgrounds or children at risk for ADHD or ASD symptomatology (Diamond and Lee, 2011; Li-Grining, 2012). Interventions focusing on older children usually try to improve these skills in those already experiencing certain difficulties, evident in low academic performance, behavioural difficulties, and social/communication problems. Even though children considered disadvantaged seem to benefit the most from such EF/SR interventions, it is unclear which specific (or a combination of) child characteristics are the most important and can lead to the best outcomes. It is important to note that the majority of studies directly and exclusively targeting EF/SR stimulation fail to report durable effects. Some training programmes lack transferable results; for instance, training one EF component does not result in improving other cognitive components (van Houdt et al., 2019; see meta-analyses by Kassai et al., 2019 and Melby-Lervåg and Hulme, 2013) and fail to generalise to academic outcomes (e.g., Banales et al., 2015; van der Donk et al., 2015). Other interventions produce short-term training-specific effects that, unfortunately, do not generalise and decline shortly after the intervention is completed (see meta-analyses by Melby-Lervåg et al., 2016 and Schwaighofer et al., 2015). This lack of transferable and durable effects might be attributed to such EF/SR interventions teaching specific EF/SR skill out of context and, therefore, ignoring potentially important contextual factors.

Consequently, the focus of the recent research on EF/SR development and improvement has shifted towards malleable environmental factors. Within this line of research, most studies have focused on parent-child interaction and home setting, and have shown that positive interactions can optimally support and promote the quality of children's EF/SR (e.g., Devine et al., 2016; Sosic-Vasic et al., 2017). In order to efficiently add to the parenting effect, more recently, researchers have shifted their attention to the role of the school/classroom environment as an important developmental context to effectively deal with children's cognition and behaviour, and teachers' role in promoting EF/SR development (e.g., de Wilde et al., 2015). Children interact with their teachers on the classroom level (i.e., teacher-student interaction—TSI) and on the dyadic (i.e., teacher-student relationship—TSR) level.

Regarding relationships between teacher and student at the classroom level, a significant contribution was made by Hamre and Pianta (2007), who introduced a Teaching Through Interactions framework. This framework places TSI as a cornerstone for student learning. The proposed framework organises TSI into three domains reflecting distinct features of these interactions; namely, emotional support (for instance, acknowledging children's emotions and experiences, and sensitively responding to them), classroom organisation (for instance, clarifying the rules and expectations), and instructional support (for instance, asking open-ended questions) (Downer et al., 2010; Hamre et al., 2013). In agreement with the framework, numerous observational studies have shown a correlation between the quality of TSI at the classroom level (i.e., emotional, instructional, and organisational support) and children's EF/SR skills (e.g., Cadima et al., 2015b; Crockett et al., 2017; Acar et al., 2018; Goble et al., 2019; for a review, see Cumming et al., 2019). For instance, a recent meta-analysis (Vandenbroucke et al., 2017, 2018) informs further regarding the strength of the correlations between these concepts, and overall effect sizes indicate small to medium associations between TSI and working memory, and a small association with inhibition, but not cognitive flexibility. However, mixed results are evident regarding positive TSI and associated gains in EF/SR performance and their components. For instance, (Hamre et al., 2013) reported that high-quality classroom organisation was related to higher inhibitory control skills in children, while, somewhat counterintuitively, they showed that high-quality emotional support was related to lower inhibitory control skills compared to children in low-quality classrooms. Despite the importance of TSI on EF/SR performance and development, there is evidence that the benefits of high-quality TSI at the classroom level are conditional on children's individual relationships with their teachers (Nguyen et al., 2020).

Regarding relationships at the dyadic level, several theories have been proposed to understand these relations. Self-determination theory, initially proposed by Deci and Ryan (1985), highlights three needs of the student in the classroom; namely, competence, autonomy, and relatedness. Classroom practises, as well as positive interactions with teachers, fostering feelings of competence, autonomy, and relatedness are likely to result in student motivation required for learning and academic success (Ryan and Pintrich, 1997). Furthermore, self-efficacy theory (Bandura, 1977) suggests that the teachers serve as role models, as students develop a range of social behaviours and communication skills by watching the teacher perform these skills. This theory highlights the importance of feedback and encouragement from teachers in relation to student performance. Finally, attachment theory (Bowlby, 1969) proposes that the teacher acts as a “secure base,” allowing the student to feel safe when making mistakes and comfortable when faced with academic challenges. Here, the focus lies on the affective components of the relationship between teacher and specific student; more specifically, closeness, conflict, and dependency (Koomen et al., 2012; Verschueren and Koomen, 2012). Closeness refers to the degree of warmth, security, and open communication. Conflict refers to negative, unpredictable, and coercive teacher-student relationships. Dependency refers to the developmentally inappropriate degree of reliance and possessiveness in the relationship. The interest for TSR at the individual level emerged rather recently; however, it consistently indicates that a positive TSR and affective teacher behaviours are (longitudinally) associated with improved child's SR abilities (Liew, 2010), more engagement (Engels et al., 2020), more profit from instruction (Crosnoe et al., 2010), improved cognitive processing (Ahnert et al., 2013), and school achievement (for meta-analyses see Roorda et al., 2011, 2017). The studies by Nguyen et al. (2020) and Crosnoe et al. (2010) suggest that children's learning and cognitive development (including EF/SR) will not benefit from high TSI quality when TSR is characterised by low levels of closeness or high levels of conflict. Moreover, research on behavioural problems associated with poor EF/SR indicates that children in need of such interventions are at an increased risk to develop conflictual relationships with their teachers (Sutherland and Oswald, 2005; de Wilde et al., 2015), thus hindering the chances of high-need children to profit from classroom quality and EF/SR intervention. A handful of studies have examined independent or additive effects of classroom-level TSI and dyadic relationship quality, but a paucity of research has examined interactional and conditional effects (for exceptions, see Crosnoe et al., 2010; Nguyen et al., 2020).

Thus, previous literature on the association between TSI/TSR and EF/SR reports somewhat inconsistent results regarding which aspects of TSI/TSR affect which components of children's EF/SR. Even though these studies already provide indications on the role TSI/TSR plays in the development of EF/SR, they are correlational in nature; thus, the causality question remains unanswered.

In light of these gaps in the current research, the purpose of the current study was to provide an overview of the existing research on the interventions focusing on improving preschool and primary school children's EF/SR performance with or without direct manipulation of TSI/TSR by means of a systematic review. Comparing the insights of diverse manipulations and training programmes (further referred to as interventions) targeting EF and SR development by activating TSI/TSR are essential for understanding how such interactions can positively influence children's development.

The following research goals guided this review:

1. To assess whether school-/class-wide interventions are effective and whether it depends on the type of manipulation (i.e., dyadic vs. classroom-level) and component(s) being activated (e.g., instructional vs. emotional support);
2. To compare and contrast the effects of these interventions on EF and SR, and their distinct components.

## Method

### Search Strategy

We have conducted a systematic literature review, which was pre-registered through PROSPERO under the number: CRD42020153324.

We have applied a multimodal search strategy to collect peer-reviewed articles, using the following techniques:

(a) Searches in databases using various combinations of key terms: (1) EF and SR, and their distinct components (and their variations as referred to in the relevant literature, for instance, ‘executive processing'), (2) preschool and primary school (and their variations, such as ‘young child'), (3) TSI and TSR, and their distinct components (and commonly associated interventions, for instance, ‘Tools of the Mind' and ‘Chicago School Readiness Project'), and (4) intervention component (and the synonyms employed, for instance, ‘stimulation').
(b) Forward and backward citation searching (i.e., searching articles that cite included articles and articles cited by the included articles).
(c) Checking grey literature by making inquiries through social networks (e.g., LinkedIn, Twitter) to the researchers in the field to locate any other unpublished, ongoing work.

We have performed a literature search of articles using Web of Science, PubMed, and ERIC databases. All English-language articles published before January 2021 were evaluated. A complete list of search terms entered in the databases is provided as Supplementary Material (Annex 1).

Articles found in the databases were first screened by the lead author at the title level to determine if the articles were relevant for this study (e.g., excluding reviews and meta-analyses). The remaining articles were then included/excluded based on the abstract. The study was included if the inclusion criteria were met at the abstract level or the abstract contained insufficient information to determine inclusion/exclusion. The full-text reading was performed on the included studies and again assessed based on the inclusion criteria of the full-text. The criteria for abstract-level and full-text inclusion/exclusion are described below. The tables used for abstract-level and full-text-level inclusion/exclusion are added as Supplementary Material (Annex 2).

The included articles were then used for a backward and forward search. For the backward search, the reference list of included studies was scanned. The titles, the abstracts, and then full-text articles, if needed, were compared to predetermined eligibility criteria. Studies that met inclusion criteria were added to the sample of studies. For the forward search, we have used the Web of Science database ‘cited by' function to identify all papers referring to one of the papers already selected. These papers were then compared to the eligibility criteria and included in the review, if appropriate.

The search and selection of the studies were conducted by the first author. The third and fourth authors each have checked 10% of the unique articles identified through the databases based on the search terms, and the results from the three researchers were then compared. There was an original agreement of 94% in the screening phase and 98% for the full-text reading. In the screening phase, disagreement between both authors mainly arose (1) when deciding whether the abstracts contained insufficient information to make an informed decision and, therefore, should be included at the abstract level, or (2) whether the abstracts did not contain relevant information and, therefore, should be excluded at the abstract level. At the full-text reading, disagreement was mainly due to the study not including the measurement or the score of the target variable for this review. Studies for which there was a disagreement were discussed further until an agreement was reached.

### Inclusion and Exclusion Criteria

Studies included at the abstract-level (1) aimed at improving EF/SR (or their distinct components) of either preschool or primary school children in regular education (3) with an intentional manipulation of the TSR at the dyadic and/or TSI at the classroom level by (4) implementing an intervention.

The selected studies at the full-text had to (1) include a measure for EF/SR (or the specific component) either pre- and post-intervention measure, or post-intervention measure for both experimental and control groups. The age of the participants had to (2) fall within the range of 3–12 years (generally corresponding to the beginning of preschool and the end of primary school). Due to the teachers in special education already receiving more training and guidance on specific difficulties/deficits experienced by the children, including such schools would not be indicative of what is offered in regulation classrooms; thus, we only focused on regular education. To increase power, we included studies with a mixed sample of typically developing children and children with a clinical profile (e.g., diagnosed with or at-risk for attention-deficit/hyperactivity disorder or autism spectrum disorder). However, the sample should regardless reflect the general population. Included articles had to (3) incorporate an intervention in their study, focusing on either children's EF/SR development or on improving the TSR/TSI and, in turn, influencing EF/SR skills. Finally, (4) studies in which participants were allocated to a programme at random (i.e., RCT), and in which participants were divided into an intervention and comparison control group without random assignment (i.e., a quasi-experimental design) or no control group was recruited but participants were assessed pre- and post-intervention (i.e., non-experimental design), were included to ensure high-quality research and strong internal validity. Of all the articles, 18 met the inclusion criteria and were reviewed (i.e., coded and analysed).

### Sample

The searches across all three databases resulted in 2,496 unique articles. The database search resulted in the inclusion of 12 original studies. These were then used for a backward and forward search, which resulted in 6 additional articles. Inquiries in the grey literature (i.e., through social networks, such as Linkedin and Twitter) for ongoing and/or unpublished work resulted in no responses and, therefore, the search did not produce any additional studies. The authors of the studies excluded at full-text reading due to missing outcome scores were contacted in an attempt to retrieve this data. However, most of the authors did not provide a response, or the response was not in accordance with the inclusion criteria (i.e., the post-intervention score was not collected).

For the included studies, statistical data were extracted or requested from the author(s). For 15 of these studies, sufficient statistical information was collected to calculate an effect size.

### Data Collection and Preparation

Data were extracted from the included studies. The variables coded were as follows:

1. Article characteristics including author, title, publication year and journal name.
2. Participant demographics including the number of participating children and teachers, grade, type of education, age of both children and teachers, gender of both children and teachers, country of data collection, and some additional characteristics (if provided), such as the education and experience of teachers, academic achievement of children, socio-economic status of the participating children/parents were documented.
3. Study characteristics were noted; specifically, data on study quality, including the design employed, the analysis performed, and the measurement tools used (by applying the GRADE approach; Schünemann et al., 2003). The measures for classroom-level TSI and dyadic-level TSR were recorded. EF/SR (and either of their components) measures were noted. When different measures were combined in a composite score, it was categorised as an overall EF/SR measure. The type of measurement (test, questionnaire, observation), the name of the instrument, and the rater were documented.
4. Intervention characteristics, including type and goal of the intervention, setting, duration, frequency, and phases of the intervention were reported, administrant of the intervention and materials used were recorded.

All selected articles were coded twice, first by the first author and second by the fourth author. The coding scheme was heavily inspired by the coding scheme used in a previous review conducted in this research team (i.e., Vandenbroucke et al., 2017, 2018). It was minimally adjusted by the first and fourth authors noting important aspects mentioned in the individual studies and adding relevant information to the coding scheme. However, not all information coded was used in subsequent data analyses. Some disagreements arose during the coding process, mainly due to the absence of the required outcome measurement/score (i.e., pre- and post-intervention or treatment vs. control post-intervention). Disagreements were handled by double-checking the coding scheme of the articles for consensus and by further discussing apparent inconsistencies; after double-checking and the discussion, an agreement was reached for all selected articles. The coding scheme used is provided as Supplementary Material (Annex 3).

### Data Analyses

First, a qualitative analysis of the systematic review is reported, providing the characteristics of the individual studies and their findings.

From the 18 studies, 15 studies reported an effect size or provided enough information to calculate the effect size based on means and standard deviations. For the remaining three studies, the effect sizes or means and standard deviations were requested from the authors but were not acquired.

For the calculation of combined effect sizes (i.e., standardised mean difference), fixed- and random-effects models can be used. Fixed-effects models assume that all studies are replications of each other and are used to compute the common effect size for the identified population (and not to generalise to other populations). Random-effects models assume that the studies are a selection of a population of studies and allow for each study to introduce its own heterogeneity. Fixed-effects models are calculated when at least two studies are available, while random-effects models are calculated when at least five studies are available (Borenstein et al., 2010). Given the characteristics and heterogeneity of the included studies (i.e., targeting various EF/SR components and diverse age groups), random-effects models are the most appropriate for the current data. Unfortunately, given the overall number of studies included in the review, the criteria for the random-effects models are not met, and, therefore, meta-analyses could not be conducted.

## Results

### Study Selection and Study Characteristics

**Figure 1:**
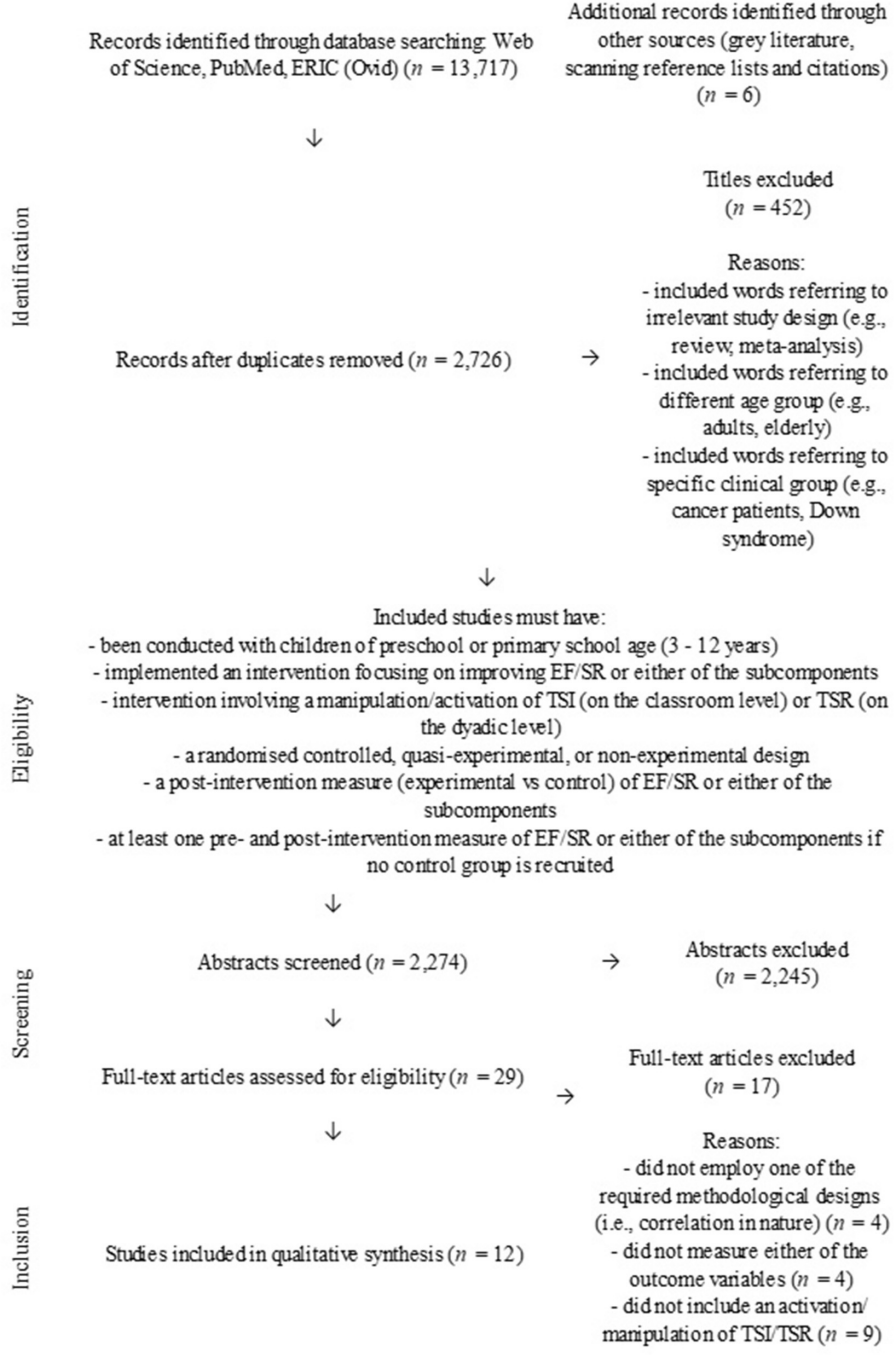
Study identification, screening, and inclusion/exclusion process.

The number of studies screened at each stage of the selection process and the reasons for exclusion are shown in Figure 1. Eighteen studies have been included in the review, and an overview of their characteristics is provided in Table 1. The studies were carried out in eight countries: Australia (*n* = 1), Belgium (*n* = 1), Chile (*n* = 1), Denmark (*n* = 1), Ghana (*n* = 3), Jamaica (*n* = 1) and United Kingdom (*n* = 1), with most being conducted in the United States (*n* = 9). Publication dates ranged from 2008 to 2020.

**Table 1:** Summary of characteristics of all studies included in the review (*n* = 18).

**PICO variable**	**Study characteristics**
Population	Age range: 3-12 years Preschool/kindergarten (*n* = 7) Primary/elementary school (*n* = 7) Mixed sample (*n* = 4)
	Status: Children with identified EF/SR difficulties (*n* = 1) Children considered at-risk for emotional/behavioural difficulties (*n* = 1) Sample included children with learning difficulties or disability (*n* = 2) Studies included high of children from low SES (*n* = 9) Children with no particular disadvantage (*n* = 5)
Intervention	Intervention approach:
	EF (*n* = 8)
	SR (*n* = 10)
	Direct TSI intervention (*n* = 14)
	Indirect TSI intervention (*n* = 4)
	focus on TSI (*n* = 18):
	- instructional support (*n* = 9)
	- emotional support (*n* = 3)
	- classroom organisation (*n* = 5)
	- combination (*n* = 1)
Comparison	Study design: RCT (*n* = 15) Quasi-experimental design (*n* = 2) Non-experimental design (*n* = 1)
	Control group conditions: Active control groups (*n* = 1) Semi-active control groups (*n* = 6) No intervention control groups (*n* = 10) No controls (*n* = 1)
Outcomes	Working memory: the Working Memory Rating Scale (Alloway et al., 2008) the Automated Working Memory Assessment (Alloway, 2007) the Corsi task backward (Milner, 1971)
	Inhibitory control: the Pencil-tapping task (adapted from Diamond & Taylor, 1996) the Preschool Learning Behaviour Scale (McDermott et al., 2002)
	Measurement of SR: the Head-Toes-Knees-Shoulders task (Cameron Ponitz et al., 2008)- the Preschool Self-Regulation Assessment (Smith-Donald et al., 2007)
	Attention regulation: d2Test of Attention (Brickenkamp, 1962, 2002)
	Behaviour regulation: the Behaviour Rating Inventory of Executive Function Teacher Form (Gioia et al., 2000)
	Emotion regulation: the Multiple Option Observation System for Experimental Studies (Tapp et al., 1995) the School Readiness and Conduct Problems: Coder Observation of Adaptation-Revised (Werthamer-Larsson et al., 1990) the Task Orientation Questionnaire (adapted from Smith-Donald et al., 2007) items drawn from the Early Development Instrument (Janus and Offord, 2007), the Teacher Observation of Child Adaptation (Werthamer-Larsson et al., 1991), and the Social Competence Scale, Teacher Version (Conduct Problems Prevention Research Group (CPPRG), 1990)
	Measurement of TSR/TSI: TSR: the Young Children's Appraisals of Teacher Support (Mantzicopoulos and Neuharth-Pritchett, 2003) the Student-Teacher Relationship Scale (Pianta, 2001)
	TSI: the Classroom Assessment Scoring System (Pianta et al., 2008) the Teacher Instructional Practices and Processes System-Primary School Version (Seidman et al., 2018)

*SES, socio-economic status; EF, executive function; SR, self-regulation; TSI, teacher-student interaction; RCT, randomised controlled trial; TSR, teacher-student relationship.*.

#### Population

To be included in the review, studies had to be conducted with children in preschool and/or primary school. In the final study selection, participants ranged from preschool to fifth grade; however, most studies did not report the chronological age of the sample, therefore, preventing an accurate calculation of the mean age of the participating children. Out of the included studies, one study specifically targeted children with working memory difficulties, one study focused on children at-risk for emotional and behavioural problems, two studies included a percentage of children with psychosocial difficulties or a disability. However, no study directly compared children with a clinical profile in relation to their typically developing peers. Nine studies included a high percentage of (or specifically targeted) children from low socio-economic backgrounds. The remaining five studies did not provide any sample specifications and are, therefore, considered as including typically developing children.

#### Intervention

In this review, the included studies have implemented EF/SR interventions in the classroom context with manipulation of the TSI/TSR. All the included studies involved a manipulation of TSI: instructional support (*n* = 9), emotional support (*n* = 3), classroom organisation (*n* = 5), or a combination of two or more aspects (*n* = 1). Fourteen studies primarily focused on improving TSI as a primary outcome and, in turn, children's consequent EF/SR as a secondary outcome (i.e., direct TSI manipulation). In comparison, four studies primarily focused on improving EF/SR outcome variables by providing teachers with TSI strategies to be implemented in their classrooms (i.e., indirect TSI manipulation). Given the focus of the study, all interventions can be broadly classified into two types: those focused on improving EF and those—on improving SR. Within these two types, the interventions are further divided based on the specific EF/SR component being targeted.

#### Comparison

Fifteen studies were randomised controlled trials, two employed a quasi-experimental design, and one—non-experimental design. Consequently, based on GRADE evaluation, fifteen studies were considered of high methodological quality, two—moderate, and one study of low quality. From the included studies, only one study used an active control group (i.e., general information technology didactics course vs. teacher-targeted classroom management intervention), one study manipulated control condition to a certain extent (i.e., training only condition vs. training + consultation/coaching), and five studies recruited additional experimental groups (e.g., comparing two intervention approaches) in addition to control group. Ten studies used a business-as-usual approach for the control group; one study did not recruit a control group.

#### Outcomes

Eight studies assessed measures of executive function components: working memory (*n* = 3), inhibition (*n* = 2), two studies assessed both working memory and inhibition, and one study examined inhibition and cognitive flexibility. Ten studies assessed measures of self-regulation: overall measure (*n* = 4), attention regulation (*n* = 1), behaviour regulation (*n* = 1), emotion regulation (*n* = 3), and one study examined behaviour and emotion regulation. Although all studies intended to manipulate TSI/TSR, only 12 studies directly measured their concept of manipulation. Out of these studies, 10 studies measured TSI on the classroom level, one study assessed TSR on the dyadic level, and one study evaluated both aspects. The main findings of each study and the evidence for each identified intervention approach are summarised below.

### Outcomes of Individual Studies

The outcomes of each study grouped into EF and SR interventions are described below. Due to some studies focusing on more than one component, the summaries for each EF/SR component are provided at the end of the intervention section. Cohen's d was used as the effect size for comparing pre- and post-intervention performance or between treatment and control group performance. As suggested by Cohen (1988), 0.2 is considered a small effect size, 0.5 represents medium effect size, and 0.8—a large effect size.

### Summary of EF Interventions

#### Working Memory

Davis et al. (2013) employed ‘Memory Mates'—a classroom-based intervention on improving attention and working memory. This intervention provided third-grade students with eight working memory strategies (in the form of visual prompt cards) for students to use during a mathematics lesson. The teacher was provided with information on working memory, typical working memory difficulties/failures, and explicit instructions for using these strategies. This study, therefore, aimed to improve working memory performance by manipulating *instructional support* provided by the teacher. This small-scale intervention led to increased on-task behaviour in all participants. Teachers provided positive comments about the effectiveness of the strategies by observing the students being more focused and needing less prompting from the teacher. Students themselves stated finding the strategies helpful and reported using at least three strategies during each target lesson. However, this study recruited only four students, highlighting the need for a larger-scale study to determine the real impact that the intervention might have on students' working memory performance.

Elliott et al. (2010) employed two contrasting classroom-based interventions aimed at improving working memory in children (i.e., 5/6 and 9/10 years of age) identified as having working memory difficulties. One of the interventions (i.e., “WM intervention”) taught teachers about the concept of working memory and provided them with guidance on how to modify educational environments to be more sensitive to the needs of children with identified difficulties. The second intervention (i.e., Direct Instruction intervention) focused on *instructional support* and provided teachers with basic principles of the direct instruction approach illustrated with case study material, in addition to working memory training described above. Neither of the two interventions resulted in improved working memory scores. However, the authors concluded that most of the participating teachers were sensitive to the needs of their students with working memory difficulties and were implementing appropriate strategies in their classrooms spontaneously; therefore, the students were already benefiting from such support.

(Vandenbroucke et al., 2017) aimed to identify contextual factors which can hinder or promote working memory performance in children from first and second grade. This study manipulated *emotional support* provided by the teacher and examined its potential effect on working memory while controlling for TSR aspects (closeness/warmth and conflict). The Cyberball paradigm was used to induce stress, and children were then presented with either a neutral message or a supportive message from a stranger, parent, or teacher. Even though the researchers did not find the expected negative effect of stress on working memory performance, they did report that teacher's emotional support improved children's working memory performance but only in those most vulnerable (i.e., children with a negative parent-child relationship). Therefore, concluding that further interventions should take into consideration the teacher-student relationship for improving children's performance and preventing working memory deficits.

#### Inhibitory Control

Ansari and Pianta (2018) explored the impacts of the MyTeachingPartner (MTP) coaching intervention on preschool children's inhibitory control. The MTP intervention aims to improve TSI by supporting teacher's abilities to provide higher quality instruction (i.e., *instructional support*) and, in turn, translate into improvements in children's school success. The authors reported a small positive effect—the MTP coaching intervention resulted in improved inhibitory control, but only for the children enrolled in classrooms with less age diversity, which is particularly important in preschool as such classrooms often contain children of different ages.

Pianta et al. (2017) evaluated the impacts of two interventions aimed to improve TSI in preschool children: the MTP coaching intervention (i.e., *instructional support*), with teachers receiving continuous, targeted feedback for improving TSI, and a semester-long course focused on effective interactions. The course aimed to increase teachers' knowledge about the role TSI plays in children's learning and teach skills for observing and labelling effective TSI. The findings reveal that children whose teachers received the MTP coaching intervention demonstrated greater gains in inhibitory control performance (i.e., small positive effect).

#### Working Memory and Inhibitory Control

Wolf et al. (2018) employed the Quality Preschool for Ghana (QP4G) programme designed to improve classroom quality and children's school readiness. The training focused on novel teaching practises that could be integrated into teacher's instructional content. This study aimed to improve working memory and inhibitory control of preschool children who were attending schools in most disadvantaged districts in Ghana. The intervention increased the levels of emotional support and behaviour management observed in the classroom. However, no effect of teacher's *instructional support* was found on children's EF outcomes.

Wolf et al. (2019) explored the QP4G intervention impacts on the same sample 1 year later. This study reported that the effects of QP4G on children's overall school readiness were sustained; however, on the domain level, no effect of teacher's *instructional support* on children's working memory and inhibitory control was found in the subsequent year.

#### Inhibitory Control and Cognitive Flexibility

Wolf (2019) employed the QP4G intervention 2 years after the baseline measurement on primary school children (i.e., in first and second grades at the follow-up). This study focused on children's inhibitory control and cognitive flexibility, as well as their behaviour regulation. At the follow-up, marginally significant impacts of teacher's *instructional support* on children's inhibitory control (small effect) and statistically significant impacts on children's cognitive flexibility and behaviour regulation (small effects) were found.

In sum, the effect of instructional support on *working memory* ranged from no to a positive effect. However, the positive effect was found in a small-sample study and should be interpreted carefully. Teacher's emotional support positively affected children's working memory; however, only in children considered most vulnerable (the effect size could not be determined based on insufficient information provided on this subsample). Similarly, the effect of instructional support on *inhibitory control* ranged from no to a small positive effect. Finally, there was a small effect of instructional support on *cognitive flexibility*. However, only one study targeted this subcomponent.

### Summary of SR Interventions

#### Overall SR

Baker-Henningham et al. (2019) investigated a violence prevention programme and its effect on first-grade children's SR. The intervention led to a decrease in teacher's use of violence against the children, increases in the emotional quality of the classroom environment, and, most importantly, teacher's *emotional support* led to benefits (i.e., small positive effect) in children's SR skills.

Connor et al. (2010) hypothesised that individualised student instruction (ISI) intervention focusing on teacher planning and *classroom organisation* would improve first graders' SR performance. Overall, teachers in the intervention group showed improved instructional and behavioural support. No significant effect was evident on children's SR score gains for students with typical SR performance in the intervention group. However, for those scoring in the lowest quartile, the ISI intervention positively affected their SR score gains. This effect ranged from small to large depending on the teacher's use of assessment-to-instruction (A2i) software for planning and individualising instruction.

Yoshikawa et al. (2015) designed the Un Buen Comienzo intervention targeting vocabulary and early literacy development in preschool children. Teachers were trained in instructional strategies (i.e., *instructional support*) and coached to implement these strategies in their classrooms. The intervention improved classroom quality, as evident in significant positive effects for emotional support, instructional support, and classroom organisation aspects. This study reported a small positive intervention effect on children's SR.

#### Attention Regulation

Keilow et al. (2019) evaluated the effectiveness of teacher-targeted Inclusive and Appreciative Classroom Management (IACM) intervention on first-grade children's selective attention. Teachers participating in the intervention received instructions on managing and positively reinforcing appropriate student behaviour through classroom rules and regulation (i.e., *classroom organisation*). Furthermore, the course taught how to use non-verbal elements in communication with students, and how this strategy can support classroom management. This study identified a small-sized treatment effect of teacher's organisational support on selective attention in intervention children.

#### Emotion Regulation

Chuang et al. (2020) employed the Incredible Years (IY) Teacher Classroom Management intervention targeting children's (i.e., from preschool to third grade) emotion (dys)regulation. The findings showed that children with higher baseline aggression benefited from teacher's *classroom organisation* more than children in the control group. More specifically, baseline aggression levels at baseline moderated the main effect on children's emotion (dys)regulation post-intervention.

Murray et al. (2018) focused on improving emotion regulation in children (i.e., preschool to second grade). Teachers participated in the IY intervention (i.e., *classroom organisation*), however, the training led to a limited change in teacher practises, potentially due to teachers' high initial levels of classroom management skills. Subsequently, no statistically significant effects on children's outcomes followed.

Webster-Stratton et al. (2008) aimed to improve first-grade children's emotional regulation by employing IY intervention and Child Social and Emotion curriculum (i.e., Dinosaur School). Results indicate a small positive effect—teachers in the intervention group used more positive classroom management strategies (i.e., *classroom organisation*) and their students, in turn, showed improved emotional regulation. Overall, children at initial risk (i.e., low levels of school readiness and high levels of conduct problems) benefitted the most from the intervention.

#### Behaviour Regulation

Cappella et al. (2012) introduced BRIDGE intervention integrating aspects of Links to Learning (L2L; Atkins et al., 2008) and MyTeachingPartner (MTP; Pianta et al., 2008). The intervention aimed to promote effective *emotional support and classroom organisation*, supportive teacher-student relationships, and preschool and primary school (i.e., preschool through fifth grade) children's social, behavioural, and academic adjustment. This study found no significant effect on children's teacher-reported behavioural regulation. However, this intervention did improve TSR closeness in children in the intervention group, but no effect was found for relationship conflict.

Jones et al. (2013) examined the Chicago School Readiness Project (CSRP) designed to support young children's development of SR by fostering emotionally close and positive relationships with teachers (i.e., *emotional support*). The results show that the CSRP intervention improved TSR on the dyadic level, and, in turn, children demonstrated significantly greater behaviour regulation skills (i.e., small positive effect).

#### Emotion and Behaviour Regulation

Daunic et al. (2013) developed Social-Emotional Learning Foundations (SELF), providing teachers with opportunities to promote emotional and behavioural SR of preschool children while teaching early literacy skills (i.e., *instructional support*). The results indicate a small-sized treatment effect that SELF improved teacher-reported behaviour regulation in children at risk for emotional and behavioural disorders.

In sum, all three types of TSI show a small positive effect on children's *overall SR* performance, with this effect being the strongest in children scoring the lowest at baseline. Findings from one study indicate a small positive effect of classroom organisation on children's *attention regulation*. For *emotion regulation*, teacher's instructional support did not result in an effect on children's performance. Teacher's classroom organisation led to a small positive effect, which was the largest for the children at initial risk. Regarding *behaviour regulation*, teacher's emotional support ranged from no to small indirect positive effect; this effect was found by teacher's emotional support improving TSR and, in turn, children's behaviour regulation. Teacher's instructional support led to a small-sized effect in children at risk for emotional and behavioural disorders. Teacher's classroom organisation did not result in an effect on children's behaviour regulation.

### Overall Summary

Taking together the findings from all interventions, the results vary from no to large positive effects, with the majority of studies reporting small positive effects. Compared to controls, all three types of TSI support seem to improve EF/SR performance in children: emotional support resulted in no to a small effect, instructional support—consistently showed a small positive effect, and classroom organisation—ranged from no to a large positive effect. The overview of the included studies and their findings can be consulted in Table 2.

**Table 2:** The overview of the included studies and their findings.

**Study author and year**	**Status**	**Age/grade**	**Total sample size**	**Intervention**	**Target outcome**	**Measure**	**TSI manipulation**	**Design**	**Control**	**Level of methodological quality**	**Results**	**Effect size (*d*)**
Ansari and Pianta (2018)	TD (7.52% children with disability)	M = 4.16 (SD = 0.48)	*n* = 1,407	“MyTeachingPartner”	Inhibitory control	Pencil-tapping task	Direct: instructional support	RCT	BAU	High	Positive effect on inhibitory control only in those with less age diversity	0.29 - small effect
Baker-Henningham et al. (2019)	TD	first Grade	*n* = 220	IRIE Classroom Toolbox	SR	Preschool Self-Regulation Assessment	Direct: emotional support	RCT	BAU	High	Positive effect on SR	n/m
Cappella et al. (2012)	TD (99% low-SES)	M = 8.0 (SD = 1.99)	*n* = 364	BRIDGE	Behaviour regulation	BRIEF	Direct: emotional support and classroom organisation	RCT	Semi-active control group (training only)	High	No positive effect on teacher-reported behavioural SR	n/a
Chuang et al. (2020)	TD (61% low-SES, 9% received special education services)	Preschool to third grade	*n* = 1,817	“Incredible Years”	Emotion regulation	TOCA-C	Direct: classroom organisation	RCT	BAU	High	Positive effect on emotion regulation moderated by baseline aggression	n/m
Connor et al. (2010)	TD (in 4 out of 10 schools, >82% low-SES)	First grade	*n* = 445	“Individualized Student Instruction”	SR	Head-Toes-Kness-Shoulders	Direct: classroom organisation	RCT	BAU	High	Positive effect on SR	0.19–0.96 - ranging from small to large effect
Davis et al. (2013)	TD	M = 8.5 years	*n* = 4	“Memory Mates”	Working memory	Working Memory Rating Scale	Indirect: instructional support	Non-experimental	No control group	Low	Positive effect on on-task behaviour	Insufficient information provided for the calculation of the effect size
Daunic et al. (2013)	Children with behavioural risk	preschool	*n* = 57	“Social-Emotional Learning Foundations”	Emotion and behaviour regulation	BRIEF	Indirect: instructional support	Quasi-experimental	BAU	Moderate	Positive effect on teacher-reported behavioural SR	0.31 - small effect
Elliott et al. (2010)	Children with WM difficulties	5/6-year-olds and 9/10-year-olds	*n* = 256	WM and “Direct Instruction” interventions	Working memory	Automated Working Memory Assessment	Direct: instructional support	Quasi-experimental	Two intervention groups and BAU control group	Moderate	No positive effect on working memory	n/a
Jones et al. (2013)	TD (predominantly low-SES, 63% single-parent)	M = 49.4 months (SD = 8.0)	*n* = 467	“Chicago School Readiness Programme”	SR	PSRA	Direct: emotional support	RCT	BAU	High	Positive effect on SR	0.03 - small indirect effect
Keilow et al. (2019)	TD	M = 7.99 years (SD = 0.39)	*n* = 1,160	“Inclusive and Appreciative Classroom Management”	Selective attention	The d2 Test of Attention	Direct: classroom organisation	RCT	Active control group (general IT didactics course)	High	Positive effect on selective attention	0.26 - small effect
Murray et al. (2018)	TD (57% low-SES, 11.4% received special education services)	preschool to second grade	*n* = 1,192	“Incredible Years”	Emotion regulation	R-TSCS Social Competence	Direct: classroom organisation	RCT	BAU	High	No positive effect on emotion regulation	n/a
Pianta et al. (2017)	TD	preschool	*n* = 2,283	“MyTeachingPartner”	Inhibitory control	Pencil-tapping task	Direct: instructional support	RCT	BAU	High	Positive effect on inhibitory control	0.24 - small effect
Vandenbroucke et al. (2017)	TD (six children diagnosed with ADHD and three - ASD)	M = 7 years 6 months	*n* = 170	Stress Induction	Working memory	Corsi task – backward	Indirect: emotional support	RCT	Two control groups and two treatment groups	High	Positive effect on working memory only in those most vulnerable	n/m
Yoshikawa et al. (2015)	TD	M = 53.5 months (SD = 3.7 months)	*n* = 1,876	“Un Buen Comienzo”	SR	Adapted version of EDI and TOCA-R	Indirect: instructional support	RCT	BAU	High	Positive effect on SR	0.16 - small effect
Webster-Stratton et al. (2008)	TD (59% low-SES)	Preschool to first grade	*n* = 1,768	“Incredible Years”	Emotion regulation	MOOSES and COCA-R	Direct: classroom organisation	RCT	BAU	High	Positive effect on emotional SR	0.38 - small effect
Wolf et al. (2019)	TD (schools from most disadvantaged districts)	M = 5.2 (preschool)	*n* = 3,345	“Quality Preschool for Ghana”	Working memory and inhibitory control	Forward digit span and Head-Toes task	Direct: instructional support	RCT	Two intervention groups and BAU control group	High	No positive effect on working memory and inhibitory control	n/a
Wolf et al. (2018)	TD (schools from most disadvantaged districts)	M = 5.2 (preschool)	*n* = 3,345	“Quality Preschool for Ghana”	Working memory and inhibitory control	Forward digit span and Head-Toes task	Direct: instructional support	RCT	Two intervention groups and BAU control group	High	Positive effect on composite EF	0.11 - small effect
Wolf (2019)	TD (schools from most disadvantaged districts)	M = 7.8 (first to second grade)	*n* = 2,421	“Quality Preschool for Ghana”	Cognitive flexibility, inhibitory control, and behaviour regulation	Dimensional Change Card Sort, Number Stroop Task, and Preschool Self-Regulation Assessment-Assessor Report	Direct: instructional support	RCT	Two intervention groups and BAU control group	High	Positive effects on cognitive flexibility, inhibitory control, and behaviour regulation	0.12, 0.08, 0.15, respectively - small effects

*TD, typically-developing; SES, socio-economic status; WM, working memory; EF, executive function; SR, self-regulation; RCT, randomised controlled trials; BAU, business-as-usual; n/a, not applicable (no effect, no d reported/calculated); n/m, not mentioned/not possible to calculate*.

## Discussion

This study aimed to systematically examine (1) the effectiveness of interventions manipulating TSI/TSR on (2) preschool and primary school children's EF/SR performance.

### Teacher-Student Interaction and Teacher-Student Relationship

The first key research goal was to assess the causal effect of TSI/TSR interventions or EF/SR interventions with an added manipulation of TSI/TSR for achieving improvements in children's EF/SR development. Even though all three types of TSI support seemed to improve EF/SR performance in children, some differences are evident. Emotional support showed at most a small effect; however, it was the type of support studied least extensively (only three studies exclusively manipulated emotional support). Instructional support was most commonly utilised (i.e., nine studies) and showed, somewhat consistently, a small positive effect of teacher's support on children's EF/SR outcomes. Organisational support appeared to be most promising although results are quite dispersed, ranging from no to a large positive effect. Only one study from those reviewed has employed two types of support in their intervention and reported no effect. Interventions directly comparing three types of support on children's outcomes would be highly informative. For instance, there is some evidence that classroom organisation is the most important support for children's behaviour regulation, while no significant influence of emotional support is found (Rimm-Kaufman et al., 2009).

Somewhat surprisingly, no study manipulated TSR at the dyadic level. Only two studies assessed TSR and showed that improving TSI led to higher quality TSR and, in turn, gains in children's EF/SR performance. However, the relationship between TSI and TSR is more likely to work in the opposite direction. Previous research suggests that improving TSR on the dyadic level will lead to improved TSI on the classroom level, and those with initially low-quality TSR tend to benefit less from improved TSI (e.g., Cadima et al., 2015a). This relationship can be explained by the notion that the children might be less open to the support provided by the teacher on the classroom level if, on the dyadic level, the relationship with the teacher is of poor quality (e.g., Spilt et al., 2012). More studies exploring TSR and its causal effect on children's EF/SR performance are needed; different aspects of this relationship (i.e., closeness, conflict, and dependency) should be assessed separately and compared. Importantly, this relationship can be measured from both the teacher's and the child's perspectives, providing a more reliable indication of the ‘real' quality of the relationship and avoid informant bias (either under- or overestimation). Once TSR is explored in more detail, combining TSI and TSR studies would be of the utmost value. Interactions between TSR and TSI could inform us which aspects of TSR and/or TSI, as well as the combination of which, leads to improved EF/SR performance.

It is important to note that the terminology used in this review does not cover all potential aspects of TSI and TSR. Based on the alternative theories, other components (e.g., autonomy) might also play a role in a successful relationship between the teacher and the student (e.g., Núñez and León, 2015). However, even though literature on TSR is grounded in numerous lines of research, its original framework seems to be most strongly influenced by the attachment theory (Riley, 2010; Sabol and Pianta, 2012), and most commonly referred to in current TSR/TSI literature (e.g., Cadima et al., 2015b). Research indicates that affective components seem to be of high importance, especially in preschool and primary school children's relationships. TSI research, consistently referring to the Teaching Through Interactions framework (Hamre and Pianta, 2007), also stems partially from the attachment theory (Downer et al., 2010), with this theory guiding most of the work on emotional support.

### Executive Function and Self-Regulation

The second research goal was to compare and contrast the effects of these interventions for EF and SR, and their distinct components. In summary, all targeted EF and SR components are potentially trainable and mutable by the interventions manipulating TSI. SR, when compared to EF, seems to be affected more strongly; however, more studies targeted SR and, therefore, might have led to somewhat inflated results. Within the distinct components, no component appears to be most positively affected. However, some of these effects (e.g., on inhibitory control) should be interpreted carefully. This aspect was targeted in studies focusing on preschool children. Preschool being the period when inhibition seems to develop most rapidly, these improvements might be partly attributed to the development rather than TSI manipulation (Liu et al., 2015).

It is important to note that the categorisations used in this review do not cover all the aspects of EF and SR. Higher-order components, like planning, and problem-solving, might be more susceptible to change if targeted directly and, therefore, should be explored as it was not under investigation in this review. Some studies employing teacher-led interventions report positive results on children's higher-order cognitive processes (e.g., Diamond, 2017), with instructional support from the teacher having the greatest potential for improving problem-solving skills.

Finally, combining which aspects of TSI/TSR affect which components of children's EF/SR is especially informative as one type of support can be very effective for one component of EF/SR but not the other (e.g., Rimm-Kaufman et al., 2009). Our results do not provide sufficient information to make such conclusions, although they indicate that instructional support seemed to be most effective on children's behaviour regulation and inhibition, while classroom organisation led to the largest gains in self-regulation, especially emotion regulation. Emotional support had the least impact but seemed to affect working memory positively. Importantly, the bidirectionality of this relationship should be explored as children's low EF/SR might lead to children being more disruptive, failing to pay attention in class, which can, in turn, affect the relationship the teacher has with the child; as a result, negatively impacting effective and appropriate stimulation of these children (e.g., Sameroff and Mackenzie, 2003). Furthermore, teachers tend to report less conflict and child dependency, and more closeness when interacting with the children who show high levels of academic and cognitive performance (see a meta-analysis by Nurmi, 2012). Therefore, it is important to assess this two-way relationship further and explore whether children's EF/SR influences their relationship with the teacher, which, in turn, can either stimulate or hinder subsequent EF/SR development.

## Suggestions for Future Research

The current study shows that there is some evidence showing the positive influence and importance of TSI on children's EF/SR. However, additional research is needed to get more insight into which teacher's behaviours should be stimulated in order to promote different components of EF/SR and consequent children's academic performance. In addition to the suggestions mentioned above, we provide some additional guidance for future research on this topic.

### Child Characteristics

The strongest intervention effects were found for the children who were considered vulnerable and disadvantaged. These children included children from low-socioeconomic backgrounds, children with poor parent-child relationships, and children with initially weakest EF/SR skills. This is consistent with the literature of direct EF/SR interventions showing the highest gains for these groups (Rimm-Kaufman, 2002; Hamre and Pianta, 2005; Baker, 2006; Merritt et al., 2012).

Other important child characteristics, such as age and gender, should be explored further. In the current review, only one study (Elliott et al., 2010) included two distinct groups: one of the preschoolers and one of the children of primary school age. However, this study found no effect on either group, therefore, failing to inform us on the role age might play in this association. This is somewhat surprising as previous literature on EF/SR interventions indicates higher gains in younger children when compared to older children (Diamond and Lee, 2011). A meta-analysis by Vandenbroucke et al. (2018) on TSI and EF showed a reverse effect—stronger associations between TSI and EF for samples with older children (within the range of 2–7 years of age).

Furthermore, child gender should also be examined as there are indications that boys tend to benefit more from the interventions when compared to girls. On the other hand, boys seem to have more conflict and less closeness in their relationships with teachers when compared to girls (e.g., Baker, 2006). However, there appears to be no research systematically examining how boys and girls separately are affected by EF/SR interventions and the role TSI/TSR plays depending on gender. Importantly, a combination of child characteristics (e.g., boys from low-SES backgrounds) should be assessed further as this can inform on the characteristics that put children most at risk, as well as of the factors that seem to act as protective mechanisms and reduce the negative impacts of such risk factors.

### Teacher Characteristics

Teacher characteristics, such as years of teaching experience or education level, can prove to be important for TSI and TSR (Cantrell and Kane, 2013; Ferguson et al., 2015; Ansari and Pianta, 2018). Through more experience and knowledge, teachers might already be equipped to apply (or already applying) different strategies in their teaching. These teachers might also be more open to change introduced through the interventions and employ the training received most effectively and efficiently. One reviewed study (Elliott et al., 2010), which found no effect of teacher's instructional support on children's working memory, has concluded that recruited teachers seemed to already use such strategies in their classrooms; therefore, the intervention did not introduce new aspects in their teaching, while children were already benefitting from such support, thus showing no additional gains post-intervention. This further suggests that studies should monitor and control what takes place in the comparison (i.e., business-as-usual) conditions; lack of substantial difference/change introduced by the intervention could, in turn, explain small effects found in reviewed studies.

### Parent/Family Characteristics

Furthermore, parent characteristics, similarly to the teacher, play a role in children's EF/SR development (Sosic-Vasic et al., 2017) and subsequent relationships (Veríssimo et al., 2017). Parenting style, parental involvement, parent's stress levels, and the parent-child relationship seem to influence children's attachment and relationship with other important figures in a child's life (e.g., O'Connor et al., 2016). One of the included studies found that children with poor parent-child relationships tend to benefit the most from the EF/SR intervention with TSI manipulation (Vandenbroucke et al., 2017). More specifically, children with high levels of conflict and low levels of warmth in their relationship with the parent were more susceptible to the teacher's support, indicating that teacher can compensate for the lack of support received from the parent. As Fay-Stammbach et al. (2014) conclude, numerous parent characteristics can act as risk or protective factors moderating the association with EF/SR, suggesting that children's EF/SR performance is, indeed, susceptible to environmental influences.

### Intervention Characteristics

Even though it was not directly explored in the current study, intervention characteristics are valuable when informing on the most effective and efficient interventions for improving EF/SR in children. Generally, the more intensive (in frequency and duration) interventions lead to the best outcomes and have the potential for longer-lasting results (Diamond and Ling, 2016; Markussen-Brown et al., 2017). However, long and demanding interventions might be difficult for the teachers to implement together with their usual classroom curriculum (e.g., Blok et al., 2005). Currently, EF/SR interventions differ markedly in duration, setting, frequency, and activities implemented and materials/measured used (Diamond, 2013). More parametric studies should be conducted in order to compare and find the most optimal length, frequency, and intensity for these interventions to be more easily implemented and still lead to the best outcomes in children's performance.

### Study Characteristics

In the current review, study characteristics were coded. Specifically, data on the underlying methodology (with RCTs ranked as of the highest quality) was noted, as well as numerous factors that may decrease the quality of the study. The limitations in the design, indirectness of evidence, and probability of publication bias were assessed. Most studies reviewed proved to be of high quality, according to the GRADE approach, and, therefore, produce reliable results. No differences in effect sizes were found based on the study quality. Nevertheless, these characteristics should be taken into account when designing further studies, and the researchers should aim for the highest quality in order to provide valid and reliable findings.

## Limitations

This systematic literature review was pre-registered through PROSPERO under the number CRD42020153324 on April 3rd 2020. However, due to a limited amount of studies found, we have expanded our study focus. Namely, we included preschool children in addition to primary school children, and we broadened our target outcome from solely focusing on improving a core EF component (i.e., working memory performance) to EF and SR (and their distinct components). The current review thus clearly shows that research on the influence of TSI for preschool and primary school children's EF/SR development and performance has emerged only recently and that the number of studies available is still limited or not easily accessible. This study also demonstrates the complexity TSI/TSR and EF/SR concepts, with some aspects of TSI/TSR and EF/SR being more commonly examined, whereas others—ignored. Thus, making it difficult to efficiently compare these types of support and judge which one might prove most promising and most effective for EF/SR development. This results in fragmentation of the literature, making it challenging to draw reliable comparisons and strong overall conclusions. Concerning TSI/TSR, no studies manipulating interactions at the dyadic level were identified. This is somewhat surprising, as the importance of TSR for children's development is evident in numerous observational studies.

A systematic literature review was conducted to provide a concise overview of the existing research on this topic. Our research focus and subsequent term selection and abstract-level inclusion/exclusion criteria were based on the Teaching Through Interactions framework (Hamre and Pianta, 2007) and attachment theory (Bowlby, 1969). Given the available literature, we believe these are the most relevant and important approaches on the TSI/TSR topic. However, due to various terms in the literature referring to TSI and TSR, as well as EF and SR (and their components), this might have led to not capturing all relevant studies. We have attempted to identify any other relevant studies through grey literature searches; however, no studies emerged. Due to high heterogeneity in concepts and operationalisation, and a relatively small overall number of studies included, it was not possible to effectively compare the level of interaction (i.e., TSIvs.TSR), types of support (e.g., instructional supportvs.classroom organisation), and different child characteristics (i.e., age and gender) through the means of a meta-regression; instead, narrative analysis was provided.

## Conclusion

Overall, the current study provided an overview of the research examining the causal effect between TSI and children's EF/SR performance, and show a positive effect between these concepts. These findings indicate that teachers can effectively promote these important cognitive processes in children. Furthermore, the current study concludes that manipulating TSI is particularly important and beneficial for children considered vulnerable or disadvantaged, suggesting that cognitive deficits can be minimised if children are supported appropriately. Further research is needed to examine which specific teacher behaviours are important for which EF/SR components and whether this is influenced by child, teacher, and parent/family characteristics. As Diamond and Ling (2016, p. 43) conclude, “each aspect of ourselves affects, and is affected by, the other aspects,” thus more research is needed to understand this potentially bidirectional two-way relationship fully. Such insights can further instruct the development and implementation of lenient and effective interventions in education.

## Data Availability Statement

The original contributions presented in the study are included in the article/Supplementary Material, further inquiries can be directed to the corresponding author.

## Author Contributions

SS conducted the literature search, the study selection process, the data extraction, the interpretation of the results, and completed the writing of the manuscript. MH provided guidance, feedback, and a critical revision of the systematic review. JD wrote and submitted the Prospero application, generated a full list of search terms, and completed 10% of article checking. CX completed 10% of article checking and contributed to the data extraction. NV and EH contributed to the Prospero application and the initial article checking. DB supervises the project and provided continuous guidance, feedback, and critical revision at every step of the review process. All authors read and approved the final manuscript.

## Conflict of Interest

The authors declare that the research was conducted in the absence of any commercial or financial relationships that could be construed as a potential conflict of interest.

## Publisher's Note

All claims expressed in this article are solely those of the authors and do not necessarily represent those of their affiliated organizations, or those of the publisher, the editors and the reviewers. Any product that may be evaluated in this article, or claim that may be made by its manufacturer, is not guaranteed or endorsed by the publisher.
