# Supplementary material for: Strengthening Executive Function and Self-Regulation Through Teacher-Student Interaction in Preschool and Primary School Children: A Systematic Review
Source: Front Psychol. 2021 Aug 19;12:718262. doi: 10.3389/fpsyg.2021.718262 (PMC8417378; doi:10.3389/fpsyg.2021.718262)
Supplement: Supplementary file 1 [file Table_1.DOCX]

Annex 1. Lists of search terms entered in the databases.

**Full list of search terms (preschool)**

1. (“Self-regulat*” OR “Executive function*” OR “cognitive control” OR “executive control”) AND (teacher* (student* OR child* OR pupil*) (relationship* OR interaction*)) AND ((prekindergarten OR pre-K OR kindergarten OR preschool OR young child* OR toddler OR preschool child*) OR (intervention* OR program* OR train* OR curricul* OR therap* OR treatment OR stimulation OR remediation))
2. (“Self-regulat*” OR “Executive function*” OR “cognitive control” OR “executive control”) AND (teacher* (student* OR child* OR pupil*) (relationship* OR interaction*)) AND ((prekindergarten OR pre-K OR kindergarten OR preschool OR young child* OR toddler OR preschool child*) OR (“Tools of the Mind” OR “Jungle memory” OR CogMed OR Montessori OR “Promoting Alternative Thinking Strategies” OR PATHS OR “Chicago School Readiness Project” OR CSRP))
3. (“Self-regulat*” OR “Executive function*” OR “cognitive control” OR “executive control”) AND (“emotional support” OR “classroom organi*” OR “classroom manage*” OR “behavio$ral support” OR “instructional support”) AND ((prekindergarten OR pre-K OR kindergarten OR preschool OR young child* OR toddler OR preschool child*) OR (intervention* OR program* OR train* OR curricul* OR therap* OR treatment OR stimulation OR remediation))
4. (“Self-regulat*” OR “Executive function*” OR “cognitive control” OR “executive control”) AND (“emotional support” OR “classroom organi*” OR “classroom manage*” OR “behavio$ral support” OR “instructional support”) AND ((prekindergarten OR pre-K OR kindergarten OR preschool OR young child* OR toddler OR preschool child*) OR (“Tools of the Mind” OR “Jungle memory” OR CogMed OR Montessori OR “Promoting Alternative Thinking Strategies” OR PATHS OR “Chicago School Readiness Project” OR CSRP))
5. (“Self-regulat*” OR “Executive function*” OR “cognitive control” OR “executive control”) AND (teacher AND (support OR closeness OR warmth OR sensitivity OR involvement OR conflict OR dependency OR structure OR affiliation OR “negative interaction” OR “positive interaction” OR “positive feelings” OR “negative feelings”)) AND ((prekindergarten OR pre-K OR kindergarten OR preschool OR young child* OR toddler OR preschool child*) OR ( intervention* OR program* OR train* OR curricul* OR therap* OR treatment OR stimulation OR remediation))
6. (“Self-regulat*” OR “Executive function*” OR “cognitive control” OR “executive control”) AND (teacher AND (support OR closeness OR warmth OR sensitivity OR involvement OR conflict OR dependency OR structure OR affiliation OR “negative interaction” OR “positive interaction” OR “positive feelings” OR “negative feelings”)) AND ((prekindergarten OR pre-K OR kindergarten OR preschool OR young child* OR toddler OR preschool child*) OR (“Tools of the Mind” OR “Jungle memory” OR CogMed OR Montessori OR “Promoting Alternative Thinking Strategies” OR PATHS OR “Chicago School Readiness Project” OR CSRP))
7. (“Cognitive processing” OR “executive processing” OR “central executive”) AND (teacher* (student* OR child* OR pupil*) (relationship* OR interaction*)) AND ((prekindergarten OR pre-K OR kindergarten OR preschool OR young child* OR toddler OR preschool child*) OR (intervention* OR program* OR train* OR curricul* OR therap* OR treatment OR stimulation OR remediation))
8. (“Cognitive processing” OR “executive processing” OR “central executive”) AND (teacher* (student* OR child* OR pupil*) (relationship* OR interaction*)) AND ((prekindergarten OR pre-K OR kindergarten OR preschool OR young child* OR toddler OR preschool child*) OR (“Tools of the Mind” OR “Jungle memory” OR CogMed OR Montessori OR “Promoting Alternative Thinking Strategies” OR PATHS OR “Chicago School Readiness Project” OR CSRP))
9. (“Cognitive processing” OR “executive processing” OR “central executive”) AND (“emotional support” OR “classroom organi*” OR “classroom manage*” OR “behavio$ral support” OR “instructional support”) AND ((prekindergarten OR pre-K OR kindergarten OR preschool OR young child* OR toddler OR preschool child*) OR (intervention* OR program* OR train* OR curricul* OR therap* OR treatment OR stimulation OR remediation))
10. (“Cognitive processing” OR “executive processing” OR “central executive”) AND (“emotional support” OR “classroom organi*” OR “classroom manage*” OR “behavio$ral support” OR “instructional support”) AND ((prekindergarten OR pre-K OR kindergarten OR preschool OR young child* OR toddler OR preschool child*) OR (“Tools of the Mind” OR “Jungle memory” OR CogMed OR Montessori OR “Promoting Alternative Thinking Strategies” OR PATHS OR “Chicago School Readiness Project” OR CSRP))
11. (“Cognitive processing” OR “executive processing” OR “central executive”) AND (teacher AND (support OR closeness OR warmth OR sensitivity OR involvement OR conflict OR dependency OR structure OR affiliation OR “negative interaction” OR “positive interaction” OR “positive feelings” OR “negative feelings”)) AND ((prekindergarten OR pre-K OR kindergarten OR preschool OR young child* OR toddler OR preschool child*) OR ( intervention* OR program* OR train* OR curricul* OR therap* OR treatment OR stimulation OR remediation))
12. (“Cognitive processing” OR “executive processing” OR “central executive”) AND (teacher AND (support OR closeness OR warmth OR sensitivity OR involvement OR conflict OR dependency OR structure OR affiliation OR “negative interaction” OR “positive interaction” OR “positive feelings” OR “negative feelings”)) AND ((prekindergarten OR pre-K OR kindergarten OR preschool OR young child* OR toddler OR preschool child*) OR (“Tools of the Mind” OR “Jungle memory” OR CogMed OR Montessori OR “Promoting Alternative Thinking Strategies” OR PATHS OR “Chicago School Readiness Project” OR CSRP))
13. (“working memory” OR “phonological loop” OR “visuospatial sketchpad” OR “short term memory”) AND (teacher* (student* OR child* OR pupil*) (relationship* OR interaction*)) AND ((prekindergarten OR pre-K OR kindergarten OR preschool OR young child* OR toddler OR preschool child*) OR (intervention* OR program* OR train* OR curricul* OR therap* OR treatment OR stimulation OR remediation))
14. (“working memory” OR “phonological loop” OR “visuospatial sketchpad” OR “short term memory”) AND (teacher* (student* OR child* OR pupil*) (relationship* OR interaction*)) AND ((prekindergarten OR pre-K OR kindergarten OR preschool OR young child* OR toddler OR preschool child*) OR (“Tools of the Mind” OR “Jungle memory” OR CogMed OR Montessori OR “Promoting Alternative Thinking Strategies” OR PATHS OR “Chicago School Readiness Project” OR CSRP))
15. (“working memory” OR “phonological loop” OR “visuospatial sketchpad” OR “short term memory”) AND (“emotional support” OR “classroom organi*” OR “classroom manage*” OR “behavio$ral support” OR “instructional support”) AND ((prekindergarten OR pre-K OR kindergarten OR preschool OR young child* OR toddler OR preschool child*) OR (intervention* OR program* OR train* OR curricul* OR therap* OR treatment OR stimulation OR remediation))
16. (“working memory” OR “phonological loop” OR “visuospatial sketchpad” OR “short term memory”) AND (“emotional support” OR “classroom organi*” OR “classroom manage*” OR “behavio$ral support” OR “instructional support”) AND ((prekindergarten OR pre-K OR kindergarten OR preschool OR young child* OR toddler OR preschool child*) OR (“Tools of the Mind” OR “Jungle memory” OR CogMed OR Montessori OR “Promoting Alternative Thinking Strategies” OR PATHS OR “Chicago School Readiness Project” OR CSRP))
17. (“working memory” OR “phonological loop” OR “visuospatial sketchpad” OR “short term memory”) AND (teacher AND (support OR closeness OR warmth OR sensitivity OR involvement OR conflict OR dependency OR structure OR affiliation OR “negative interaction” OR “positive interaction” OR “positive feelings” OR “negative feelings”)) AND ((prekindergarten OR pre-K OR kindergarten OR preschool OR young child* OR toddler OR preschool child*) OR ( intervention* OR program* OR train* OR curricul* OR therap* OR treatment OR stimulation OR remediation))
18. (“working memory” OR “phonological loop” OR “visuospatial sketchpad” OR “short term memory”) AND (teacher AND (support OR closeness OR warmth OR sensitivity OR involvement OR conflict OR dependency OR structure OR affiliation OR “negative interaction” OR “positive interaction” OR “positive feelings” OR “negative feelings”)) AND ((prekindergarten OR pre-K OR kindergarten OR preschool OR young child* OR toddler OR preschool child*) OR (“Tools of the Mind” OR “Jungle memory” OR CogMed OR Montessori OR “Promoting Alternative Thinking Strategies” OR PATHS OR “Chicago School Readiness Project” OR CSRP))
19. (“inhibitory control” OR “interference control” OR “selective attention” OR “behavio* inhibition” OR “cognitive inhibition” OR “attentional control” OR “attentional inhibition” OR “executive attention” OR “response control” OR “delay of gratification”) AND (teacher* (student* OR child* OR pupil*) (relationship* OR interaction*)) AND ((prekindergarten OR pre-K OR kindergarten OR preschool OR young child* OR toddler OR preschool child*) OR (intervention* OR program* OR train* OR curricul* OR therap* OR treatment OR stimulation OR remediation))
20. (“inhibitory control” OR “interference control” OR “selective attention” OR “behavio* inhibition” OR “cognitive inhibition” OR “attentional control” OR “attentional inhibition” OR “executive attention” OR “response control” OR “delay of gratification”) AND (teacher* (student* OR child* OR pupil*) (relationship* OR interaction*)) AND ((prekindergarten OR pre-K OR kindergarten OR preschool OR young child* OR toddler OR preschool child*) OR (“Tools of the Mind” OR “Jungle memory” OR CogMed OR Montessori OR “Promoting Alternative Thinking Strategies” OR PATHS OR “Chicago School Readiness Project” OR CSRP))
21. (“inhibitory control” OR “interference control” OR “selective attention” OR “behavio* inhibition” OR “cognitive inhibition” OR “attentional control” OR “attentional inhibition” OR “executive attention” OR “response control” OR “delay of gratification”) AND (“emotional support” OR “classroom organi*” OR “classroom manage*” OR “behavio$ral support” OR “instructional support”) AND ((prekindergarten OR pre-K OR kindergarten OR preschool OR young child* OR toddler OR preschool child*) OR (intervention* OR program* OR train* OR curricul* OR therap* OR treatment OR stimulation OR remediation))
22. (“inhibitory control” OR “interference control” OR “selective attention” OR “behavio* inhibition” OR “cognitive inhibition” OR “attentional control” OR “attentional inhibition” OR “executive attention” OR “response control” OR “delay of gratification”) AND (“emotional support” OR “classroom organi*” OR “classroom manage*” OR “behavio$ral support” OR “instructional support”) AND ((prekindergarten OR pre-K OR kindergarten OR preschool OR young child* OR toddler OR preschool child*) OR (“Tools of the Mind” OR “Jungle memory” OR CogMed OR Montessori OR “Promoting Alternative Thinking Strategies” OR PATHS OR “Chicago School Readiness Project” OR CSRP))
23. (“inhibitory control” OR “interference control” OR “selective attention” OR “behavio* inhibition” OR “cognitive inhibition” OR “attentional control” OR “attentional inhibition” OR “executive attention” OR “response control” OR “delay of gratification”) AND (teacher AND (support OR closeness OR warmth OR sensitivity OR involvement OR conflict OR dependency OR structure OR affiliation OR “negative interaction” OR “positive interaction” OR “positive feelings” OR “negative feelings”)) AND ((prekindergarten OR pre-K OR kindergarten OR preschool OR young child* OR toddler OR preschool child*) OR ( intervention* OR program* OR train* OR curricul* OR therap* OR treatment OR stimulation OR remediation))
24. (“inhibitory control” OR “interference control” OR “selective attention” OR “behavio* inhibition” OR “cognitive inhibition” OR “attentional control” OR “attentional inhibition” OR “executive attention” OR “response control” OR “delay of gratification”) AND (teacher AND (support OR closeness OR warmth OR sensitivity OR involvement OR conflict OR dependency OR structure OR affiliation OR “negative interaction” OR “positive interaction” OR “positive feelings” OR “negative feelings”)) AND ((prekindergarten OR pre-K OR kindergarten OR preschool OR young child* OR toddler OR preschool child*) OR (“Tools of the Mind” OR “Jungle memory” OR CogMed OR Montessori OR “Promoting Alternative Thinking Strategies” OR PATHS OR “Chicago School Readiness Project” OR CSRP))
25. (“cognitive flexibility” OR “set* shift*” OR “attention* shif*” OR “task* switch*” OR “mental flexibility” OR “fluency” OR “generativity”) AND (teacher* (student* OR child* OR pupil*) (relationship* OR interaction*)) AND ((prekindergarten OR pre-K OR kindergarten OR preschool OR young child* OR toddler OR preschool child*) OR (intervention* OR program* OR train* OR curricul* OR therap* OR treatment OR stimulation OR remediation))
26. (“cognitive flexibility” OR “set* shift*” OR “attention* shif*” OR “task* switch*” OR “mental flexibility” OR “fluency” OR “generativity”) AND (teacher* (student* OR child* OR pupil*) (relationship* OR interaction*)) AND ((prekindergarten OR pre-K OR kindergarten OR preschool OR young child* OR toddler OR preschool child*) OR (“Tools of the Mind” OR “Jungle memory” OR CogMed OR Montessori OR “Promoting Alternative Thinking Strategies” OR PATHS OR “Chicago School Readiness Project” OR CSRP))
27. (“cognitive flexibility” OR “set* shift*” OR “attention* shif*” OR “task* switch*” OR “mental flexibility” OR “fluency” OR “generativity”) AND (“emotional support” OR “classroom organi*” OR “classroom manage*” OR “behavio$ral support” OR “instructional support”) AND ((prekindergarten OR pre-K OR kindergarten OR preschool OR young child* OR toddler OR preschool child*) OR (intervention* OR program* OR train* OR curricul* OR therap* OR treatment OR stimulation OR remediation))
28. (“cognitive flexibility” OR “set* shift*” OR “attention* shif*” OR “task* switch*” OR “mental flexibility” OR “fluency” OR “generativity”) AND (“emotional support” OR “classroom organi*” OR “classroom manage*” OR “behavio$ral support” OR “instructional support”) AND ((prekindergarten OR pre-K OR kindergarten OR preschool OR young child* OR toddler OR preschool child*) OR (“Tools of the Mind” OR “Jungle memory” OR CogMed OR Montessori OR “Promoting Alternative Thinking Strategies” OR PATHS OR “Chicago School Readiness Project” OR CSRP))
29. (“cognitive flexibility” OR “set* shift*” OR “attention* shif*” OR “task* switch*” OR “mental flexibility” OR “fluency” OR “generativity”) AND (teacher AND (support OR closeness OR warmth OR sensitivity OR involvement OR conflict OR dependency OR structure OR affiliation OR “negative interaction” OR “positive interaction” OR “positive feelings” OR “negative feelings”)) AND ((prekindergarten OR pre-K OR kindergarten OR preschool OR young child* OR toddler OR preschool child*) OR ( intervention* OR program* OR train* OR curricul* OR therap* OR treatment OR stimulation OR remediation))
30. (“cognitive flexibility” OR “set* shift*” OR “attention* shif*” OR “task* switch*” OR “mental flexibility” OR “fluency” OR “generativity”) AND (teacher AND (support OR closeness OR warmth OR sensitivity OR involvement OR conflict OR dependency OR structure OR affiliation OR “negative interaction” OR “positive interaction” OR “positive feelings” OR “negative feelings”)) AND ((prekindergarten OR pre-K OR kindergarten OR preschool OR young child* OR toddler OR preschool child*) OR (“Tools of the Mind” OR “Jungle memory” OR CogMed OR Montessori OR “Promoting Alternative Thinking Strategies” OR PATHS OR “Chicago School Readiness Project” OR CSRP))

**Full list of search terms (primary school)**

1. (“Self-regulat*” OR “Executive function*” OR “cognitive control” OR “executive control”) AND (teacher* (student* OR child* OR pupil*) (relationship* OR interaction*)) AND ((primary school child* OR young child* OR elementary school) OR (intervention* OR program* OR train* OR curricul* OR therap* OR treatment OR stimulation OR remediation))
2. (“Self-regulat*” OR “Executive function*” OR “cognitive control” OR “executive control”) AND (teacher* (student* OR child* OR pupil*) (relationship* OR interaction*)) AND ((primary school child* OR young child* OR elementary school) OR (“Tools of the Mind” OR “Jungle memory” OR CogMed OR Montessori OR “Promoting Alternative Thinking Strategies” OR PATHS OR “Chicago School Readiness Project” OR CSRP))
3. (“Self-regulat*” OR “Executive function*” OR “cognitive control” OR “executive control”) AND (“emotional support” OR “classroom organi*” OR “classroom manage*” OR “behavio$ral support” OR “instructional support”) AND ((primary school child* OR young child* OR elementary school) OR (intervention* OR program* OR train* OR curricul* OR therap* OR treatment OR stimulation OR remediation))
4. (“Self-regulat*” OR “Executive function*” OR “cognitive control” OR “executive control”) AND (“emotional support” OR “classroom organi*” OR “classroom manage*” OR “behavio$ral support” OR “instructional support”) AND ((primary school child* OR young child* OR elementary school) OR (“Tools of the Mind” OR “Jungle memory” OR CogMed OR Montessori OR “Promoting Alternative Thinking Strategies” OR PATHS OR “Chicago School Readiness Project” OR CSRP))
5. (“Self-regulat*” OR “Executive function*” OR “cognitive control” OR “executive control”) AND (teacher AND (support OR closeness OR warmth OR sensitivity OR involvement OR conflict OR dependency OR structure OR affiliation OR “negative interaction” OR “positive interaction” OR “positive feelings” OR “negative feelings”)) AND ((primary school child* OR young child* OR elementary school) OR ( intervention* OR program* OR train* OR curricul* OR therap* OR treatment OR stimulation OR remediation))
6. (“Self-regulat*” OR “Executive function*” OR “cognitive control” OR “executive control”) AND (teacher AND (support OR closeness OR warmth OR sensitivity OR involvement OR conflict OR dependency OR structure OR affiliation OR “negative interaction” OR “positive interaction” OR “positive feelings” OR “negative feelings”)) AND ((primary school child* OR young child* OR elementary school) OR (“Tools of the Mind” OR “Jungle memory” OR CogMed OR Montessori OR “Promoting Alternative Thinking Strategies” OR PATHS OR “Chicago School Readiness Project” OR CSRP))
7. (“Cognitive processing” OR “executive processing” OR “central executive”) AND (teacher* (student* OR child* OR pupil*) (relationship* OR interaction*)) AND ((primary school child* OR young child* OR elementary school) OR (intervention* OR program* OR train* OR curricul* OR therap* OR treatment OR stimulation OR remediation))
8. (“Cognitive processing” OR “executive processing” OR “central executive”) AND (teacher* (student* OR child* OR pupil*) (relationship* OR interaction*)) AND ((primary school child* OR young child* OR elementary school) OR (“Tools of the Mind” OR “Jungle memory” OR CogMed OR Montessori OR “Promoting Alternative Thinking Strategies” OR PATHS OR “Chicago School Readiness Project” OR CSRP))
9. (“Cognitive processing” OR “executive processing” OR “central executive”) AND (“emotional support” OR “classroom organi*” OR “classroom manage*” OR “behavio$ral support” OR “instructional support”) AND ((primary school child* OR young child* OR elementary school) OR (intervention* OR program* OR train* OR curricul* OR therap* OR treatment OR stimulation OR remediation))
10. (“Cognitive processing” OR “executive processing” OR “central executive”) AND (“emotional support” OR “classroom organi*” OR “classroom manage*” OR “behavio$ral support” OR “instructional support”) AND ((primary school child* OR young child* OR elementary school) OR (“Tools of the Mind” OR “Jungle memory” OR CogMed OR Montessori OR “Promoting Alternative Thinking Strategies” OR PATHS OR “Chicago School Readiness Project” OR CSRP))
11. (“Cognitive processing” OR “executive processing” OR “central executive”) AND (teacher AND (support OR closeness OR warmth OR sensitivity OR involvement OR conflict OR dependency OR structure OR affiliation OR “negative interaction” OR “positive interaction” OR “positive feelings” OR “negative feelings”)) AND ((primary school child* OR young child* OR elementary school) OR ( intervention* OR program* OR train* OR curricul* OR therap* OR treatment OR stimulation OR remediation))
12. (“Cognitive processing” OR “executive processing” OR “central executive”) AND (teacher AND (support OR closeness OR warmth OR sensitivity OR involvement OR conflict OR dependency OR structure OR affiliation OR “negative interaction” OR “positive interaction” OR “positive feelings” OR “negative feelings”)) AND ((primary school child* OR young child* OR elementary school) OR (“Tools of the Mind” OR “Jungle memory” OR CogMed OR Montessori OR “Promoting Alternative Thinking Strategies” OR PATHS OR “Chicago School Readiness Project” OR CSRP))
13. (“working memory” OR “phonological loop” OR “visuospatial sketchpad” OR “short term memory”) AND (teacher* (student* OR child* OR pupil*) (relationship* OR interaction*)) AND ((primary school child* OR young child* OR elementary school) OR (intervention* OR program* OR train* OR curricul* OR therap* OR treatment OR stimulation OR remediation))
14. (“working memory” OR “phonological loop” OR “visuospatial sketchpad” OR “short term memory”) AND (teacher* (student* OR child* OR pupil*) (relationship* OR interaction*)) AND ((primary school child* OR young child* OR elementary school) OR (“Tools of the Mind” OR “Jungle memory” OR CogMed OR Montessori OR “Promoting Alternative Thinking Strategies” OR PATHS OR “Chicago School Readiness Project” OR CSRP))
15. (“working memory” OR “phonological loop” OR “visuospatial sketchpad” OR “short term memory”) AND (“emotional support” OR “classroom organi*” OR “classroom manage*” OR “behavio$ral support” OR “instructional support”) AND ((primary school child* OR young child* OR elementary school) OR (intervention* OR program* OR train* OR curricul* OR therap* OR treatment OR stimulation OR remediation))
16. (“working memory” OR “phonological loop” OR “visuospatial sketchpad” OR “short term memory”) AND (“emotional support” OR “classroom organi*” OR “classroom manage*” OR “behavio$ral support” OR “instructional support”) AND ((primary school child* OR young child* OR elementary school) OR (“Tools of the Mind” OR “Jungle memory” OR CogMed OR Montessori OR “Promoting Alternative Thinking Strategies” OR PATHS OR “Chicago School Readiness Project” OR CSRP))
17. (“working memory” OR “phonological loop” OR “visuospatial sketchpad” OR “short term memory”) AND (teacher AND (support OR closeness OR warmth OR sensitivity OR involvement OR conflict OR dependency OR structure OR affiliation OR “negative interaction” OR “positive interaction” OR “positive feelings” OR “negative feelings”)) AND ((primary school child* OR young child* OR elementary school) OR ( intervention* OR program* OR train* OR curricul* OR therap* OR treatment OR stimulation OR remediation))
18. (“working memory” OR “phonological loop” OR “visuospatial sketchpad” OR “short term memory”) AND (teacher AND (support OR closeness OR warmth OR sensitivity OR involvement OR conflict OR dependency OR structure OR affiliation OR “negative interaction” OR “positive interaction” OR “positive feelings” OR “negative feelings”)) AND ((primary school child* OR young child* OR elementary school) OR (“Tools of the Mind” OR “Jungle memory” OR CogMed OR Montessori OR “Promoting Alternative Thinking Strategies” OR PATHS OR “Chicago School Readiness Project” OR CSRP))
19. (“inhibitory control” OR “interference control” OR “selective attention” OR “behavio* inhibition” OR “cognitive inhibition” OR “attentional control” OR “attentional inhibition” OR “executive attention” OR “response control” OR “delay of gratification”) AND (teacher* (student* OR child* OR pupil*) (relationship* OR interaction*)) AND ((primary school child* OR young child* OR elementary school) OR (intervention* OR program* OR train* OR curricul* OR therap* OR treatment OR stimulation OR remediation))
20. (“inhibitory control” OR “interference control” OR “selective attention” OR “behavio* inhibition” OR “cognitive inhibition” OR “attentional control” OR “attentional inhibition” OR “executive attention” OR “response control” OR “delay of gratification”) AND (teacher* (student* OR child* OR pupil*) (relationship* OR interaction*)) AND ((primary school child* OR young child* OR elementary school) OR (“Tools of the Mind” OR “Jungle memory” OR CogMed OR Montessori OR “Promoting Alternative Thinking Strategies” OR PATHS OR “Chicago School Readiness Project” OR CSRP))
21. (“inhibitory control” OR “interference control” OR “selective attention” OR “behavio* inhibition” OR “cognitive inhibition” OR “attentional control” OR “attentional inhibition” OR “executive attention” OR “response control” OR “delay of gratification”) AND (“emotional support” OR “classroom organi*” OR “classroom manage*” OR “behavio$ral support” OR “instructional support”) AND ((primary school child* OR young child* OR elementary school) OR (intervention* OR program* OR train* OR curricul* OR therap* OR treatment OR stimulation OR remediation))
22. (“inhibitory control” OR “interference control” OR “selective attention” OR “behavio* inhibition” OR “cognitive inhibition” OR “attentional control” OR “attentional inhibition” OR “executive attention” OR “response control” OR “delay of gratification”) AND (“emotional support” OR “classroom organi*” OR “classroom manage*” OR “behavio$ral support” OR “instructional support”) AND ((primary school child* OR young child* OR elementary school) OR (“Tools of the Mind” OR “Jungle memory” OR CogMed OR Montessori OR “Promoting Alternative Thinking Strategies” OR PATHS OR “Chicago School Readiness Project” OR CSRP))
23. (“inhibitory control” OR “interference control” OR “selective attention” OR “behavio* inhibition” OR “cognitive inhibition” OR “attentional control” OR “attentional inhibition” OR “executive attention” OR “response control” OR “delay of gratification”) AND (teacher AND (support OR closeness OR warmth OR sensitivity OR involvement OR conflict OR dependency OR structure OR affiliation OR “negative interaction” OR “positive interaction” OR “positive feelings” OR “negative feelings”)) AND ((primary school child* OR young child* OR elementary school) OR ( intervention* OR program* OR train* OR curricul* OR therap* OR treatment OR stimulation OR remediation))
24. (“inhibitory control” OR “interference control” OR “selective attention” OR “behavio* inhibition” OR “cognitive inhibition” OR “attentional control” OR “attentional inhibition” OR “executive attention” OR “response control” OR “delay of gratification”) AND (teacher AND (support OR closeness OR warmth OR sensitivity OR involvement OR conflict OR dependency OR structure OR affiliation OR “negative interaction” OR “positive interaction” OR “positive feelings” OR “negative feelings”)) AND ((primary school child* OR young child* OR elementary school) OR (“Tools of the Mind” OR “Jungle memory” OR CogMed OR Montessori OR “Promoting Alternative Thinking Strategies” OR PATHS OR “Chicago School Readiness Project” OR CSRP))
25. (“cognitive flexibility” OR “set* shift*” OR “attention* shif*” OR “task* switch*” OR “mental flexibility” OR “fluency” OR “generativity”) AND (teacher* (student* OR child* OR pupil*) (relationship* OR interaction*)) AND ((primary school child* OR young child* OR elementary school) OR (intervention* OR program* OR train* OR curricul* OR therap* OR treatment OR stimulation OR remediation))
26. (“cognitive flexibility” OR “set* shift*” OR “attention* shif*” OR “task* switch*” OR “mental flexibility” OR “fluency” OR “generativity”) AND (teacher* (student* OR child* OR pupil*) (relationship* OR interaction*)) AND ((primary school child* OR young child* OR elementary school) OR (“Tools of the Mind” OR “Jungle memory” OR CogMed OR Montessori OR “Promoting Alternative Thinking Strategies” OR PATHS OR “Chicago School Readiness Project” OR CSRP))
27. (“cognitive flexibility” OR “set* shift*” OR “attention* shif*” OR “task* switch*” OR “mental flexibility” OR “fluency” OR “generativity”) AND (“emotional support” OR “classroom organi*” OR “classroom manage*” OR “behavio$ral support” OR “instructional support”) AND ((primary school child* OR young child* OR elementary school) OR (intervention* OR program* OR train* OR curricul* OR therap* OR treatment OR stimulation OR remediation))
28. (“cognitive flexibility” OR “set* shift*” OR “attention* shif*” OR “task* switch*” OR “mental flexibility” OR “fluency” OR “generativity”) AND (“emotional support” OR “classroom organi*” OR “classroom manage*” OR “behavio$ral support” OR “instructional support”) AND ((primary school child* OR young child* OR elementary school) OR (“Tools of the Mind” OR “Jungle memory” OR CogMed OR Montessori OR “Promoting Alternative Thinking Strategies” OR PATHS OR “Chicago School Readiness Project” OR CSRP))
29. (“cognitive flexibility” OR “set* shift*” OR “attention* shif*” OR “task* switch*” OR “mental flexibility” OR “fluency” OR “generativity”) AND (teacher AND (support OR closeness OR warmth OR sensitivity OR involvement OR conflict OR dependency OR structure OR affiliation OR “negative interaction” OR “positive interaction” OR “positive feelings” OR “negative feelings”)) AND ((primary school child* OR young child* OR elementary school) OR ( intervention* OR program* OR train* OR curricul* OR therap* OR treatment OR stimulation OR remediation))
30. (“cognitive flexibility” OR “set* shift*” OR “attention* shif*” OR “task* switch*” OR “mental flexibility” OR “fluency” OR “generativity”) AND (teacher AND (support OR closeness OR warmth OR sensitivity OR involvement OR conflict OR dependency OR structure OR affiliation OR “negative interaction” OR “positive interaction” OR “positive feelings” OR “negative feelings”)) AND ((primary school child* OR young child* OR elementary school) OR (“Tools of the Mind” OR “Jungle memory” OR CogMed OR Montessori OR “Promoting Alternative Thinking Strategies” OR PATHS OR “Chicago School Readiness Project” OR CSRP))
