# Supplementary material for: Strengthening Executive Function and Self-Regulation Through Teacher-Student Interaction in Preschool and Primary School Children: A Systematic Review
Source: Front Psychol. 2021 Aug 19;12:718262. doi: 10.3389/fpsyg.2021.718262 (PMC8417378; doi:10.3389/fpsyg.2021.718262)
Supplement: Supplementary file 2 [file Table_2.DOCX]

Annex 2. Tables used for abstract-level and full-text-level inclusion/exclusion.

**Checklist (abstract)**

Check the box 'yes' if the aspect is present, check the box 'no' if the aspect is not present, check 'N/M' if the aspect is not mentioned

|  | Description | Yes | No | N/M |
| --- | --- | --- | --- | --- |
| 1. | Key concepts of the study (research question includes relevant components) |  |  |  |
| AND/OR | Inclusion: Self-regulation, emotional self-regulation, behavioural self-regulation |  |  |  |
|  | Inclusion: Executive functions, executive control, or cognitive control |  |  |  |
|  | Inclusion: Working memory, short term memory, phonological loop, visuospatial sketchpad |  |  |  |
|  | Inclusion: Cognitive flexibility, shifting |  |  |  |
|  | Inclusion: Inhibition, attentional control, response control |  |  |  |
| AND | Inclusion: Teacher-student (child) interaction (TSI) or relationship (TSR) |  |  |  |
| 2. | Target group of the study |  |  |  |
| AND/OR | Inclusion: Primary school children (i.e., grades 1-6, or aged 6-12) |  |  |  |
|  | Inclusion: Preschool children, children in prekindergarten and kindergarten (aged 3-6) |  |  |  |
| AND | Inclusion: Children in regular schools |  |  |  |
| 3. | Activities and/or interventions applied |  |  |  |
|  | Inclusion: (Part of) the manipulation directly targeting or activating TSI |  |  |  |
| AND/OR | Inclusion: Executed by teachers |  |  |  |
|  | Inclusion: Executed by trainers and/or coaches |  |  |  |
| 4. | Main findings (strength for each outcome) |  |  |  |
| 5. | Other analyses (relevant secondary outcomes) |  |  |  |
| 6. | Limitations |  |  |  |
| 7. | Implications (relevance for teachers and other professionals) |  |  |  |

If neither of the aspects of ‘1.’ **are met or mentioned** -> exclude at the abstract level

If the aspects of ‘1.’ are met, but 2-7 **are not mentioned** -> include at the abstract level

If neither of the aspects of ‘2.’ **are met** (i.e., focus on a different age group) -> exclude at the abstract level

**Checklist full-text)**

Check the box 'yes' if the aspect is present, check the box 'no' if the aspect is not present, check 'N/M' if the aspect is not mentioned

Name of the study:

Name of the (possible) intervention:

|  | Description | Yes | No | N/M |
| --- | --- | --- | --- | --- |
| 1. | Key concepts of the study of the study |  |  |  |
| AND/OR | Executive functions (EF) |  |  |  |
|  | Self-regulation (SR) |  |  |  |
|  | *Effect on cognitive flexibility* |  |  |  |
|  | *Effect on inhibition* |  |  |  |
|  | *Effect on working memory* |  |  |  |
| AND | Teacher-student (child) interaction (TSI) |  |  |  |
| 2. | Target group of the study |  |  |  |
| AND/OR | Primary school children (i.e., grades 1-6; the majority of the students are in grades 1-6, or aged 6-12) |  |  |  |
|  | Preschool children, children in prekindergarten and kindergarten (aged 3-6) |  |  |  |
| AND | Children in regular schools |  |  |  |
|  | Exclusion of children with an IQ<80 (i.e., intellectual disability) |  |  |  |
| 3. | *Theoretical justification (for the purpose of the intervention theory)* |  |  |  |
| 4. | Activities and interventions to apply |  |  |  |
| AND | Manipulation directly targeting or activating TSI (on the dyadic level, the classroom level, or a combination of both) |  |  |  |
| AND/OR | Executed by teachers |  |  |  |
|  | Executed by trainers and/or coaches |  |  |  |
| 5. | Evidence for effectiveness (Level 5 or level 4 must be present) | Yes | No | N/M |
| OR | Level 5 (RCT with follow-up measures) |  |  |  |
|  | Level 4 (norm related research, change theory research, and quasi-experimental research) |  |  |  |
|  | *Level 3 (outcome monitoring: change research, goal achievement research, client satisfaction research and research on drop out)* |  |  |  |
|  | *Level 2 (Meta-analysis, literature study, focus groups, and grounded theory/intervention mapping)* |  |  |  |
|  | *Level 1 (descriptive research, document analysis, interviews and Delphi panels)* |  |  |  |

*Items in Italic are not necessary for inclusion but can be interesting to check for a later phase of the research.*

Only if the aspects of 1, 2, 4, and 5 are met -> include at the full-text level

If EF/SR aspects of ‘1.’ are either **primary or secondary outcomes** -> include at the full-text level

If the aspects of ‘2.’ are at least **a part of the sample** -> include at the full-text

If the aspects of ‘4.’ are at least **a part of the manipulation** -> include at the full-text
