# Supplementary material for: Strengthening Executive Function and Self-Regulation Through Teacher-Student Interaction in Preschool and Primary School Children: A Systematic Review
Source: Front Psychol. 2021 Aug 19;12:718262. doi: 10.3389/fpsyg.2021.718262 (PMC8417378; doi:10.3389/fpsyg.2021.718262)
Supplement: Supplementary file 3 [file Table_3.DOCX]

| **Study information** | | | |
| --- | --- | --- | --- |
| *DOI* | |  | |
| Author(s) | |  | |
| Title | |  | |
| Publication year | |  | |
| **Sample characteristics** | | | |
| Number of children (control and experimental) | |  | |
| Number of teachers (control and experimental) | |  | |
| *Number of classes* | |  | |
| *Number of schools* | |  | |
| Regular/special education | |  | |
| Grade(s) | |  | |
| *Size of the class (number of children in class)* | |  | |
| Age of students (M and SD, in months) | |  | |
| Age of teachers (M and SD, in months) | |  | |
| Sex of students (percentages) | |  | |
| Sex of teachers (percentages) | |  | |
| Typically/atypically developing sample (e.g., ASD, ADHD, learning disabilities, behavioural problems) | |  | |
| *Teaching experience (M and SD, in years)* | |  | |
| *Education of teachers* | |  | |
| *Number of working days teachers* | |  | |
| *Urban/rural schools (percentages)* | |  | |
| Country of data collection | |  | |
| *Number of parents (control and experimental)* | |  | |
| *Age of parents (M and SD, in months)* | |  | |
| *Sex of parents (percentages)* | |  | |
| *SES (education level parents/free lunch/income/other)* | |  | |
| **Measures** | | | |
| **TSI** | | **Instrument** | **Rater** |
| Classroom-level | Emotional support |  |  |
|  | Instructional support |  |  |
|  | Classroom management |  |  |
|  | Overall classroom quality |  |  |
|  | Other |  |  |
| Dyadic-level | Closeness |  |  |
|  | Conflict |  |  |
|  | Dependency |  |  |
|  | Overall teacher-student relationship |  |  |
|  | Other |  |  |
| **EF** | | **Instrument** | **Rater** |
| Inhibitory control | |  |  |
| Working memory | |  |  |
| Cognitive flexibility | |  |  |
| Overall executive functioning | |  |  |
| Other | |  |  |
| **Self-regulation** | | **Instrument** | **Rater** |
| Behaviour regulation | |  |  |
| Emotion regulation | |  |  |
| Attention regulation | |  |  |
| Overall self-regulation | |  |  |
| Other | |  |  |
| **Academic achievement** | | **Instrument** | **Rater** |
| Mathematics | |  |  |
| Spelling | |  |  |
| Reading | |  |  |
| Overall school achievement / general ability | |  |  |
| Other (e.g., vocabulary) | |  |  |
| **Other outcomes** | | **Instrument** | **Rater** |
|  | |  |  |
| **Parent-child relationship** | |  |  |
| **Methodological design** | | | |
| Study type (e.g., experimental with a longitudinal component) | |  | |
| Blinding (y/n) | |  | |
| Study design (e.g., repeated measures; between- / within-subject; counterbalancing y/n) | |  | |
| Randomisation (e.g., simple, block, stratified) | |  | |
| **Manipulation characteristics** | | | |
| Type of manipulation | |  | |
| Main focus / goal | |  | |
| Theoretical rationale | |  | |
| Administered by | |  | |
| Materials used | |  | |
| Setting | |  | |
| Duration | |  | |
| Frequency | |  | |
| Phases (pre-manipulation, manipulation, post-manipulation/follow-up) | |  | |
| *Manipulation training administered by* | |  | |
| *Manipulation training administered to* | |  | |
| *Training administered on (i.e., content)* | |  | |
| *Training conditions* | |  | |
| Is TSI the only manipulation (y/n); if not, what else? | |  | |
| **Analyses** | | | |
| Statistical model (e.g., ANOVA, SEM, etc.) | |  | |
| *Transformations* | |  | |
| Inference criteria | |  | |
| *Data exclusion (e.g., outliers)* | |  | |
| *Missing data* | |  | |
| **Results** | | | |
| Significant differences between groups (*F*, *p* values) | |  | |
| Non-significant differences between groups | |  | |
| Significant differences within groups (*F*, *p* values) | |  | |
| Non-significant differences within groups | |  | |
| Significant differences in interaction effect (*F*, *p* values) | |  | |
| Non-significant differences in interaction effect | |  | |
| Impact of the manipulation on primary variables | |  | |
| Impact of the manipulation on secondary variables | |  | |
| Overall conclusion | |  | |
| Limitations | |  | |

**Quality of body of evidence: GRADE approach**

**Levels of quality of a body of evidence in the GRADE approach**

| **Underlying methodology** | **Quality rating** |  |
| --- | --- | --- |
| Randomized trials; or double-upgraded observational studies. | High |  |
| Downgraded randomized trials; or upgraded observational studies. | Moderate |  |
| Double-downgraded randomized trials; or observational studies. | Low |  |
| Triple-downgraded randomized trials; or downgraded observational studies; or case series/case reports. | Very low |  |

**Factors that may decrease the quality level of a body of evidence**

| 1. Limitations in the design and implementation of available studies suggesting high likelihood of bias. |  |
| --- | --- |
| 2. Indirectness of evidence (indirect population, intervention, control, outcomes). |  |
| 3. Unexplained heterogeneity or inconsistency of results (including problems with subgroup analyses). |  |
| 4. Imprecision of results (wide confidence intervals). |  |
| 5. High probability of publication bias. |  |

**Factors that may increase the quality level of a body of evidence**

| 1. Large magnitude of effect. |  |
| --- | --- |
| 2. All plausible confounding would reduce a demonstrated effect or suggest a spurious effect when results show no effect. |  |
| 3. Dose-response gradient. |  |

*Note.* Refers to grading scheme described in chapter 12 of Cochrane Manual (note that not everything is relevant, given that we are working with NRS)

**Further guidelines for factor 1 (of 5) in a GRADE assessment: Going from assessments of risk of bias to judgements about study limitations for main outcomes**

| **Risk of bias** | **Across studies** | **Interpretation** | **Considerations** | **GRADE assessment of study limitations** |
| --- | --- | --- | --- | --- |
| Low risk of bias. | Most information is from studies at low risk of bias. | Plausible bias unlikely to seriously alter the results. | No apparent limitations. | No serious limitations, do not downgrade. |
| Unclear risk of bias. | Most information is from studies at low or unclear risk of bias. | Plausible bias that raises some doubt about the results. | Potential limitations are unlikely to lower confidence in the estimate of effect. | No serious limitations, do not downgrade. |
|  |  |  | Potential limitations are likely to lower confidence in the estimate of effect. | Serious limitations, downgrade one level. |
| High risk of bias. | The proportion of information from studies at high risk of bias is sufficient to affect the interpretation of results. | Plausible bias that seriously weakens confidence in the results. | Crucial limitation for one criterion, or some limitations for multiple criteria, sufficient to lower confidence in the estimate of effect. | Serious limitations, downgrade one level. |
|  |  |  | Crucial limitation for one or more criteria sufficient to substantially lower confidence in the estimate of effect. | Very serious limitations, downgrade two levels. |
